# Mild Sol–Gel Conditions and High Dielectric Contrast: A Facile Processing toward Large-Scale Hybrid Photonic Crystals for Sensing and Photocatalysis

**DOI:** 10.1021/acsami.1c23653

**Published:** 2022-04-21

**Authors:** Simone Bertucci, Heba Megahd, Andrea Dodero, Sergio Fiorito, Francesco Di Stasio, Maddalena Patrini, Davide Comoretto, Paola Lova

**Affiliations:** † Dipartimento di Chimica e Chimica Industriale, Università degli Studi di Genova, Via Dodecaneso 31, Genova 16145, Italy; ‡ Photonic Nanomaterials, Istituto Italiano di Tecnologia, Via Morego 30, Genova 16163, Italy; § Dipartimento di Fisica, Università degli Studi di Pavia, Via A. Bassi 6, Pavia 27100, Italy

**Keywords:** sol−gel synthesis, hybrid materials, photonic crystals, sensing, light management, photocatalysis

## Abstract

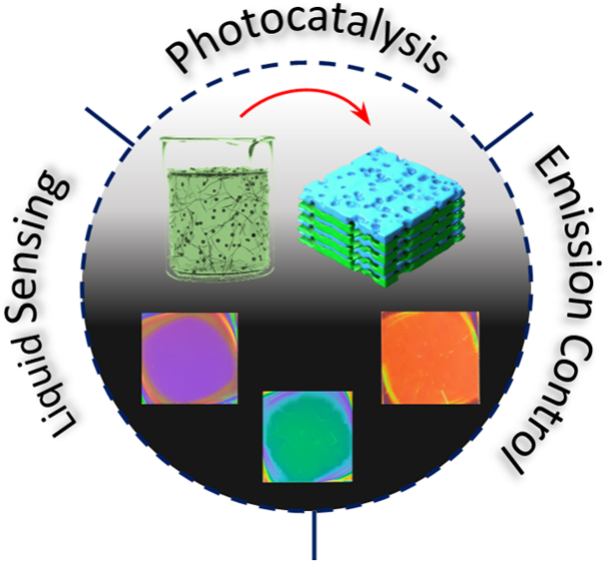

Solution processing of highly performing photonic crystals has been a towering ambition for making them technologically relevant in applications requiring mass and large-area production. It would indeed represent a paradigm changer for the fabrication of sensors and for light management nanostructures meant for photonics and advanced photocatalytic systems. On the other hand, solution-processed structures often suffer from low dielectric contrast and poor optical quality or require complex deposition procedures due to the intrinsic properties of components treatable from solution. This work reports on a low-temperature sol–gel route between the alkoxides of Si and Ti and poly(acrylic acid), leading to stable polymer–inorganic hybrid materials with tunable refractive index and, in the case of titania hybrid, photoactive properties. Alternating thin films of the two hybrids allows planar photonic crystals with high optical quality and dielectric contrast as large as 0.64. Moreover, low-temperature treatments also allow coupling the titania hybrids with several temperature-sensitive materials including dielectric and semiconducting polymers to fabricate photonic structures. These findings open new perspectives in several fields; preliminary results demonstrate that the hybrid structures are suitable for sensing and the enhancement of the catalytic activity of photoactive media and light emission control.

## Introduction

Using wet chemistry and solution processing methods for the fabrication of high dielectric contrast photonic crystals (PhCs) bearing the optical properties of sputtered inorganic ones has been a long-term aspiration in photonics in order to reduce the costs and simplify the fabrication processes. PhCs are dielectric lattices allowing the formation of forbidden bands (stop-bands) for the electromagnetic propagation in the ultraviolet, visible, and near-infrared spectral regions and find several technological applications in light control for waveguiding and lighting devices, in photon harvesting for photocatalysis and photovoltaics, and in sensing.^1^ Since their first description,^2,3^ PhCs made of high refractive index semiconductors such as GaAs, GaN, or Si coupled with air voids into complex architectures have been the paradigm for light control in optics and photonics.^4−6^ These materials indeed provide strong light confinement owing to their large dielectric contrast. On the other hand, their growth needs high-vacuum technologies which often hinder their use for large-area applications owing to the impossibility to efficiently scale up and reduce the cost of their fabrication, regardless of the geometry of the structure.^4−7^ On this matter, planar 1D PhCs such as distributed Bragg reflectors (DBRs) and microcavities processed from a solution have attracted paramount interest for their simple structures and ease of fabrication. Moreover, even though to date inorganic DBRs are still a paradigm for applications related to light control, solution processing has raised an ever-increasing interest both for potential large-area applications and for mass production, as well as for properties denied to bulky inorganics such as flexibility and permeability.^8^

Solution-processed DBRs are typically fabricated at the lab scale by alternated spin-casting of high and low refractive index polymer solutions or of colloidal suspensions of oxide particles and subsequent sintering.^1^ While polymer DBRs do not offer dielectric contrast suitable for many applications,^1^ the quality of the interfaces built with sintered nanoparticles is often poor.^1^ As a matter of fact, these two methods do not represent a solution to the problem, and several research groups are still focusing their work on the issue.^9,10^ Although systems based on nanoparticles have remained essentially unchanged in the last 2 decades, polymer planar structures evolved rapidly mainly owing to their major chemical flexibility. The first polymer DBRs were indeed made of cellulose acetate (CA) and polystyrene and displayed a refractive index contrast of Δ*n* = 0.11.^11^ Today, the largest dielectric contrast available for commercial polymer DBRs approaches Δ*n* = 0.35,^12^ a value that is still far from those needed in advanced photonics applications. On the other hand, high refractive indexes can be obtained by adding a high density of delocalized electrons with conjugated moieties or polarizable atoms in the polymer backbones. Large efforts have thus been dedicated to develop polymers with intrinsically high refractive index, including polyimides,^13,14^ highly brominated polymers,^15^ hyperbranched polysulphides,^16^ and systems containing phosphorus,^17^ which display values between 1.6 and 1.7 in the visible range. Inversely vulcanized polymers have also been reported with indexes larger than 1.9.^10,18^ Conversely, low refractive index values are obtained by inserting voids and porosity in the bulk material or using perfluoropolymers.^19,20^

Finally, in recent years, hybrid polymer–inorganic nanocomposites entered the scene of planar PhCs. These materials consist of a polymer matrix loaded with high refractive index inorganic nanoparticles such as diamond and titanium or zinc oxide.^21,22^ Structures like these allow for competitive refractive indexes,^23,24^ but high concentrations of inorganics are hindered as several constraints such as wide spectral transparency and sub-nanometric interfacial roughness are mandatory. Pristine metal oxide nanoparticle thin films have also been used together with polymer layers^25^ to achieve higher dielectric contrast or for the modification of the DBR permeability for molecular detection.^26^ Recent literature reports polymer layers alternated to V_2_O_5_,^26^ TiO_2_,^27,28^ ZnO,^29^ and CuSCN,^30^ but issues related to relatively rough interfaces and dielectric contrast are still challenging.

An alternative route to achieve solution-processed high dielectric contrast DBRs exploits sol–gel reactions of precursors of metal or metalloid oxides.^31,32^ In these processes, the oxide precursors are hydrolyzed to form a sol which is then cast by spin- or dip-coating to from dry xerogel thin films, which in turn are densified by high-temperature reactive post-deposition annealing.^31,32^ To this regard, several works have been reported on the fabrication of thin-film DBRs,^32−35^ but the large reactivity of the precursors often leads to the formation of scattering centers. Moreover, the high temperatures needed for the stabilization of the thin films, up to and exceeding 500 °C, hinder their processability and their possible coupling with temperature-sensitive materials.^32−35^ To solve this issue, polymers or small organic molecules can be used as stabilizers, also allowing the decrease of the temperature of post-deposition processes: poly(imide)–TiO_2_ hybrid thin films for transparent memory devices^36^ and mechanically performing acrylic resin–titania^37,38^ are just a few examples of similar structures. In recent years, Stingelin and co-workers^9^ reported high optical quality thin films and DBRs based on high refractive hybrid titania obtained by hydrolyzation of TiCl_4_ in cold water and subsequent mixing of the hydrated oxide product with a water solution of poly(vinyl alcohol). The hybrid sols were then cast by spin-coating or dip-coating, aged or washed in water to remove the excess HCl developed during the hydrolysis of TiCl_4_, and annealed at low temperature.^9,39^ When coupled with perfluoropolymers, these high index hybrids allowed DBRs with outstanding dielectric contrast. Similar systems also show an interesting chromic response when exposed to deep UV.^40^ However, relatively long stabilization times and the development of a large amount of HCl as a by-product of TiCl_4_ hydrolysis^9,39^ are points that need improvement. These advancements represent a solid starting point to make high-dielectric contrast sol–gel processing the new paradigm for the fabrication of functional DBRs. To gather structures with perspective applications going beyond the widely reported light mirroring/filtering, mild pH and relatively low annealing temperature are mandatory to couple high-index sol–gel materials with other low-index dielectrics and active media, including polymers and some pH-sensitive oxides themselves. Indeed, high post-deposition processing temperatures would forbid using polymer films that would degrade or simply collapse at temperature above their glass transition. Extremely low pH, instead, would affect the surface of several polymer species inducing scattering centers in the structure, dissolve several materials such as silicon oxide, and degrade conjugated polymers.

In this work, we focused on developing mild conditions in sol–gel reactions to obtain high index photoactive polymer–titania (Ti–Hy) hybrids compatible with the processing of different dielectrics and low-index polymer–silica (Si–Hy) ones. Mild pH and low post-deposition temperature are possible thanks to (i) the use of alkoxide precursors which produces a stoichiometric amount of alcohol instead of acidic species upon hydrolyzation; (ii) avoiding water as a solvent, but solubilizing the system in the alcoholic by-product of the alkoxide hydrolysis itself and running the latter with a catalytic amount of HCl; and (iii) employing a reactive polymer soluble in alcohols to control the kinetics of the sol–gel reaction and stabilize the sol and the xerogel with respect to environmental moisture. This approach also stabilized intermediate sols and gels in room condition, thus reducing the amount of polymer needed for the purpose and allowing annealing temperatures as low as 80–300 °C, which are obtained on a simple laboratory hot plate leading to high refractive index and versatile titania-based hybrid films. As a result, DBRs made with the two hybrid materials show dielectric contrast typical of inorganic DBRs and sub-nanometric interfacial roughness. Furthermore, the mild pH and temperature allow fabricating DBRs and microcavities through coupling Ti–Hy to several polymer thin films for the first time and also facilitate the integration of emitters, including organic and polymer dyes, which are commonly sensitive to low pH values and high temperatures. The process results in a high dielectric contrast compared with the literature^41^ for solution-processed DBRs. As a proof of concept, we also exploit the porosity of the hybrid films in DBR structures to adsorb molecular species in sensing and photodegradation processes where the Hy–Ti layers also demonstrate the photoactivity, while the photonic structures favor light harvesting enhancement.

## Experimental Section

### Materials

Titanium(IV) butoxide (TIBU, 97% reagent grade), tetraethyl orthosilicate (TEOS, 98% reagent grade), poly(acrylic acid) (PAA) (*M*_w_ = 1800), 1-butanol(anhydrous), and hydrochloric acid (37% v/v), purchased from Sigma-Aldrich, were employed in the preparation of Ti–Hy and Si–Hy thin films. Poly(2,6-dimethyl-1,4-phenylene oxide) (PPO, *M*_w_ = 30,000, SABIC) and poly(methyl methacrylate) (PMMA, Sigma-Aldrich *M*_w_ = 50,000) dissolved in toluene and Hyflon AD60 dissolved in Galden HT 110 (Solvay Specialty Polymers) were also used as low refractive index media alternated to the Ti–Hy films. Unless differently specified, the analytes were purchased from Sigma-Aldrich.

### Sample Preparation

For the preparation of the oxides first, sols were prepared by addition of 10 mL of a solution of PAA in butanol and a catalytic amount of HCl (100 μL) to 10 mL of the respective alkoxide. Varying the ratio between the alkoxides and PAA in the starting solutions allows controlling the volume fraction of the organic and inorganic components within the deposited films. The concentrations employed are reported in Table 1.

**Table 1:** Concentrations Employed in the Preparation of Titania and Silica Hybrids

sample	TEOS (mol/L)	TIBU (mol/L)	PAA (mg/mL)
Ti–Hy 70% v/v		1.2	7.5
Ti–Hy 92% v/v		1.5	2
Ti–Hy 97% v/v		1.5	1
Si–Hy 92% v/v	2.7		4
Si–Hy 97% v/v	2.7		2

The hydrolysis is then conducted under stirring for 2 h at room temperature for the titania hybrid and at 100 °C for 3 h for the silica one. The sols maintain transparency and processability for weeks after preparation (Supporting Information Figure S1).

Thin films and DBRs were prepared by dynamic spin-coating of the sols and of polymer solutions on glass substrates with the rotation speed ranging from 5400 to 12,000 rounds per minute. The hybrids were consequently treated on a common laboratory hot plate at temperatures ranging from 80 to 300 °C. Multilayers were grown by subsequent spin-coating of alternated high (Ti–Hy) and low (Si–Hy, PPO, PMMA, or Hyflon) refractive index media until the desired number of periods was achieved. The baking temperature for Ti–Hy/Si–Hy multilayers was 300 °C, while the temperature employed for the baking of Ti–Hy alternated to polymer layers was 80 °C. This temperature was chosen as it is below the glass-transition temperature of all the polymers employed (*T*_gPMMA_ = 85 °C, *T*_gHyflon_ = 125 °C, and *T*_gPPO_ = 215 °C), and it allows to cast the Ti–Hy layers avoiding the polymer beneath to mollify and collapse creating inhomogeneities and scattering effects. Microcavities were fabricated alternating layer of Ti–Hy and PMMA to form the first DBR. A defect layer of poly(9,9-dioctylfluorene-*alt*-benzothiadiazole) (F8BT) dissolved in toluene was then cast on the structure before the deposition of a second Ti–Hy/PMMA DBR. Two layers of CA (*n* = 1.46) were cast before and after the F8BT layer to protect it from the slightly acidic pH of the titania layers.

### Characterization Techniques

The thermal behavior of the sols as well as their weight loss was investigated by differential scanning calorimetry (DSC) and thermogravimetric analysis (TGA). The samples were prepared by airing a few milliliters of sol solution inside a Petri dish in the open air until dry and subsequent treatment under vacuum to remove moisture and excess solvent. DSC was carried out with a DSC 1 STAR from Mettler Toledo in the range of temperature between −150 and 500 °C (heating/cooling rate of 20 °C/min and nitrogen flow of 10 mL/min) using liquid nitrogen for cooling. TGA measurements were carried out using a TGA/DSC 1 STAR from Mettler Toledo in the range of temperature between 25 and 900 °C (heating/cooling rate of 10 °C/min and nitrogen flow of 80 mL/min).

Spectroscopic ellipsometry was employed to retrieve the optical functions of the hybrid material thin films cast on silicon and quartz substrates using a VASE ellipsometer by J.A. Woollam Co. Inc. Incidence angles ranging from 55 to 75° were adopted. Data analysis was performed through the dedicated software WVASE32.

Fourier transform infrared spectroscopy (FT-IR) was performed using a Vertex 70 spectrometer from Bruker in attenuated total reflection (ATR) configuration using a diamond crystal. Samples are prepared by drop-casting the hybrid sols on silicon substrates, treated thermally for different times, and then positioned facing the ATR crystal.

Multilayer reflectance and angle-resolved transmittance were performed with home-made optical setups using a y-fiber probe connected to an AvaSpec-ULS2048CL-EVO-RS spectrometer (Avantes, 200–1150 nm, resolution 1.4 nm) and a halogen-deuterium light source Micropack DH2000BAL. Angle-resolved transmittance spectra were collected for both P and S light polarizations, connecting a source and a detector to a collimation system with optical fibers. Normal incidence and angle-resolved photoluminescence spectra were collected with the same detector and exciting the material with a continuous wave laser (Oxxius 405 nm) with a power of 50 mW.

Cross-sectional scanning electron microscopy (SEM) micrographs of hybrid DBRs were collected using a JEOL JSM-7500 FA (Jeol, Tokyo, Japan) operating at an acceleration voltage of 10 kV in back-scattering configuration. Cross-sectional micrographs were collected upon freezing the samples in liquid nitrogen and subsequent breaking. Atomic force microscopy was performed in the tapping mode using a Nanosurf Core atomic force microscopy (AFM) with a resolution of 512 lines on the areas of 1 × 1 μm and 10 × 10 μm.

Transfer matrix method (TMM) modeling of the DBR optical response was performed as previously detailed,^1^ employing the refractive index from the literature and layer thickness retrieved from SEM micrographs (see the Supporting Information).

Harvesting enhancement assessment was evaluated by measuring the absorbance of methylene blue in the multilayers upon UV light irradiation. To this purpose, the samples were first immersed in an aqueous solution containing 6 ppm of the dye for 30 min in a dark environment. The samples were then irradiated with a 365 nm light emitting diode (LED), and the absorbance of the dye was collected every 5 min.

Sensing measurements were instead collected by measuring the transmittance spectra of a multilayer, immersed in a fused silica cuvette filled with the desired analyte, as recorded by an Avantes Avaspec-mini spectrometer. Performing the measurement in a cuvette in the transmittance mode allows us indeed to avoid light focusing issues associated with the variation of the environment refractive index.

## Results and Discussion

### Polymer–Inorganic Sol–Gel Reaction

**Figure 1:**
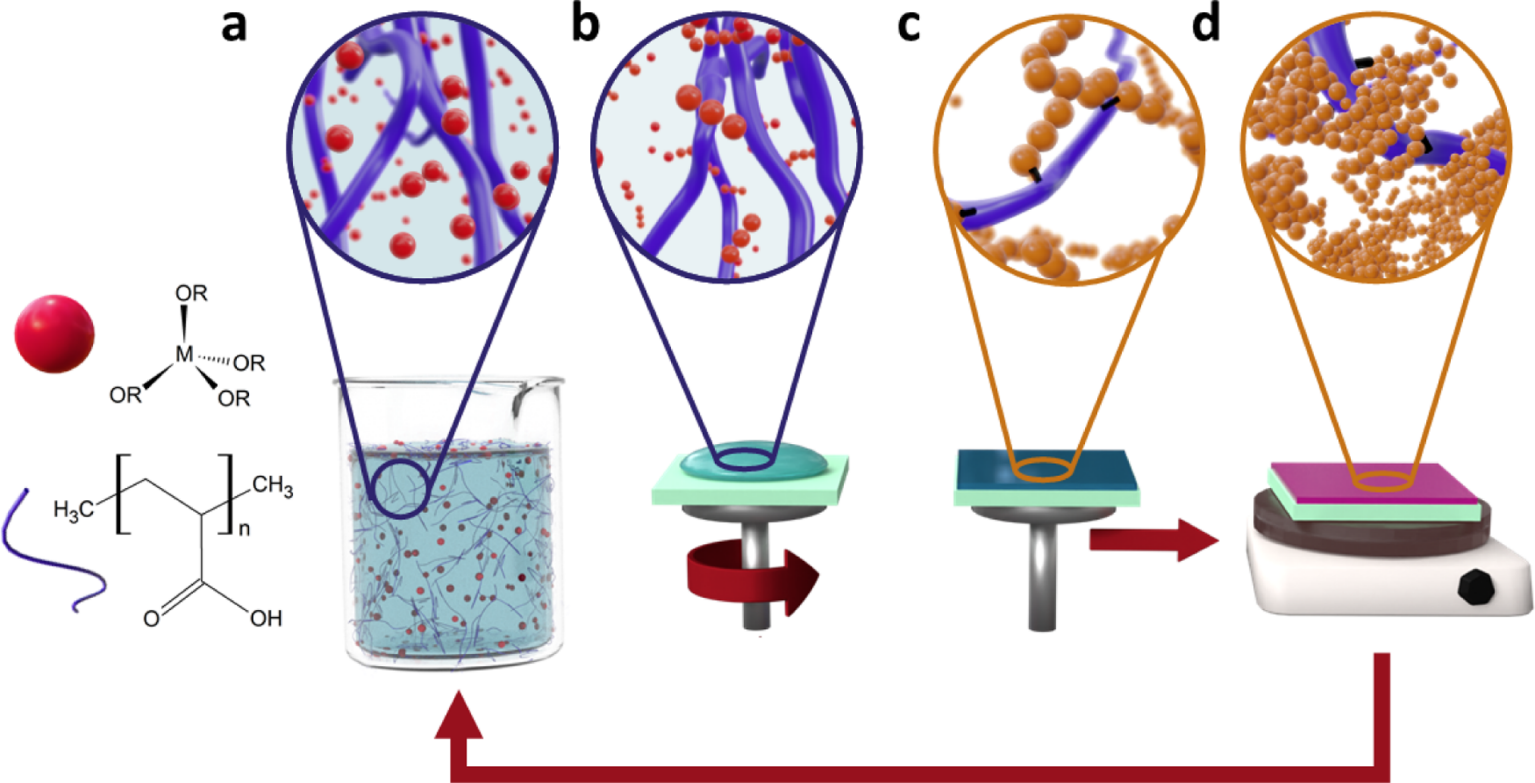
Schematic of the low-temperature sol–gel deposition process developed for the Ti–Hy and Si–Hy thin films and multilayered structures: (a) formation of sol; (b) deposition via dynamic spin-coating; (c) formation and drying of film; and (d) thermal annealing of the hybrid structure.

As illustrated in Figure 1, the method employed includes the preparation of a sol where the addition of HCl in butanol under continuous stirring triggers the hydrolyzation reaction of the alkoxide precursor and promotes condensation reactions toward the end of the oligomer chains, while limiting unwanted branching that could lead to gelation^42^ (panel a in Figure 1 and Supporting Information Scheme S1).

The solutions are stirred at room temperature for 2 h for Ti–Hy and for 3 h at 100 °C for Si–Hy before their deposition to ensure complete homogenization. The concentration of PAA in the sol was calculated as volume percentage with respect to the oxide amount considering the full conversion of the Ti and Si precursors to obtain concentrations from 70 to 97% v/v. As mentioned above, the choice of butanol as a solvent and PAA as a stabilizer was not arbitrary. In fact, butanol prevents an exchange reaction with the highly reactive TIBU, which could lead to rapid conversion and thus uncontrolled formation of titania nanoparticles which would act as scattering centers and defects in the films and in the PhCs. Moreover, a strong excess of butanol, which is also a by-product of TIBU hydrolysis (see the reaction mechanisms in the Supporting Information, Scheme S2) disadvantages the quick and strongly favored reaction. These issues are less critical with TEOS, which is more stable in room conditions and usually gels with slower kinetics. Second, butanol provides the sols with the proper viscosity and vapor tension for the spin-coating process. Regarding the polymer stabilizer, low molecular mass PAA (1800 g/mol) was adopted for multiple reasons: (i) it is completely soluble in butanol; (ii) TIBU and TEOS are already fairly viscous liquids and then low molecular mass PAA limits the increase of viscosity, which would be detrimental for the spin-coating deposition; and (iii) carboxylic functionalities, in the presence of an acid catalyst, should receive nucleophilic attack from the alkoxide, a relatively strong nucleophile.^43^

After the hydrolysis, a xerogel is formed through condensation reactions upon spin-casting of the sol (panels b and c in Figure 1). Then, the film is annealed on a simple laboratory hot plate for 60 s at a temperature ranging from 80 to 300 °C for the titania hybrid and at 300 °C for the silica one (panel d). The effect of the annealing process, mandatory for our materials, is estimated with different characterization methods (see Figures S2–S5 in the Supporting Information), highlighting the major differences between treated and non-treated films.

The final chemical structure of the system, similar to the one of several hybrids already reported in the literature,^9,36,37^ is schematized in Supporting Information Scheme S2. In this proposed structure, the inorganic matrix is stabilized by the formation of covalent bonds in a condensation reaction and by hydrogen bonds with PAA. The structure is insoluble in various organic solvents and aqueous solutions. The optimal annealing temperature was investigated by DSC and TGA (Supporting Information Figure S2). The analysis indicates a full conversion of Ti–Hy at 300 °C and of Si–Hy at about 500 °C. Nevertheless, the integration of the polymer matrix stabilizes the films even when treated at lower temperatures (see below). The optimal annealing time was instead evaluated by measuring the reflectance spectra of a thin film cast on silicon during the annealing on the hot plate (Figures S3 and S4). As the interference pattern arising from the film is a function of its optical thickness,^44^ monitoring the evolution of the pattern allows identifying about 60 s as the time required for film stabilization under the annealing conditions. Finally, FT-IR spectroscopy was used to assess the removal of butanol and butoxide from the xerogel upon heating (Figure S5). ATR spectra show that most of the organic content related to both the polymer and the solvent is removed upon 30 s annealing (C–H stretching at 2700 cm^–1^). Similarly, O–H stretching, visible as a broad peak at about 3400 cm^–1^ for both hybrids and assigned to the solvent, is also strongly reduced within the same timeframe.

### Optical Properties of the Hybrids

**Figure 2:**
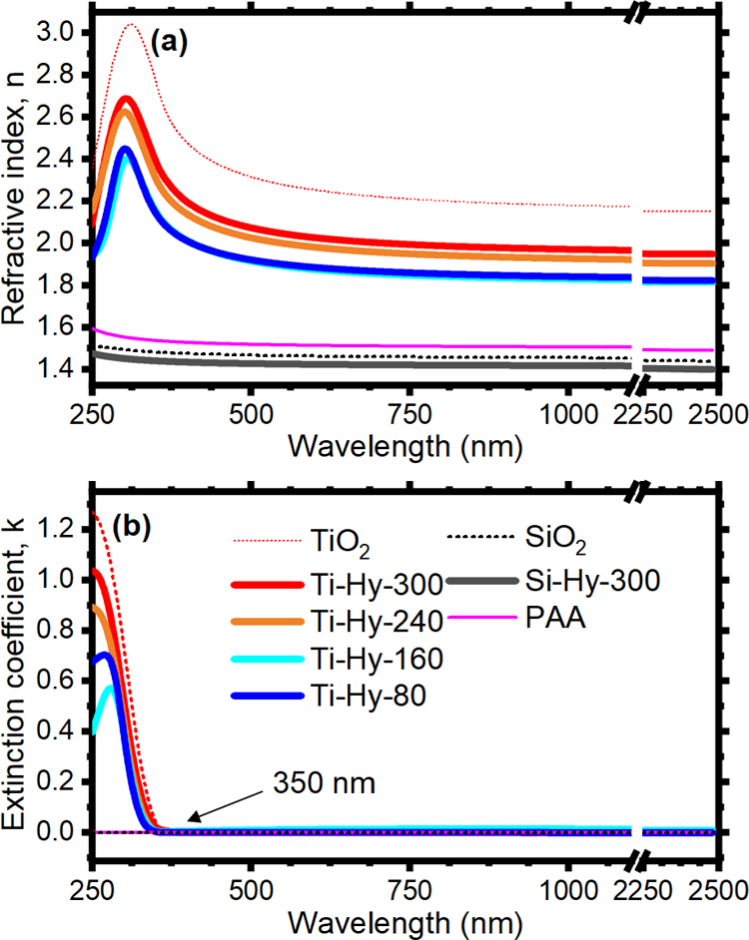
Refractive index (a) and extinction coefficient (b) spectra of thin films of Ti–Hy 97% v/v annealed at different temperatures (300 °C red, 240 °C orange, 160 °C cyan, and 80 °C blue line) and of Si–Hy 97% v/v annealed at 300 °C (gray line) as compared with the value obtained from pristine compact silica and titania reported in the literature(dotted lines)^46,47^ and with the value retrieved for PAA.

The refractive index dispersion of the hybrids was retrieved from spectroscopic ellipsometry on films spin-cast on silicon and quartz substrates and thermally annealed at different temperatures. The refractive index spectra are reported in Figure 2a, with the index of PAA,^45^ reference titania in the anatase phase,^46^ and silicon oxide^47^ for comparison. Concerning Ti–Hy, the spectral shape of the optical function resembles the one for crystalline TiO_2_ with an almost constant behavior in the near-infrared spectral range and an increase approaching the ultraviolet part of the spectrum, followed by a drop at 300 nm. The refractive index value for the Ti–Hy is a function of the annealing temperature and at λ = 550 nm reaches *n* = 2.05 at 300 °C, *n* = 2.00 at 240 °C, and *n* = 1.89 at 160 and 80 °C. These values agree with a reactive process occurring at about 300 °C (Figure S2) inducing a sudden increase of refractive index (film densification) at the higher annealing temperatures. The lower value with respect to the crystalline titania (*n* = 2.28 at 550 nm) is attributed to different factors: (i) the thin film contains a small amount of PAA, which, in agreement with the effective medium theory,^48^ decreases the index of the film and (ii) sol–gel materials are commonly porous (*n*_air_ = 1). Notice also that the refractive index of the materials treated at the different temperatures is very stable over time (Figure S6). Moreover, it is also possible to tune the refractive index of the material by inserting larger amounts of the PAA stabilizer in the sol (see Figure S7).

Regarding the Si–Hy films, we characterized only those treated at 300 °C. That is due to the mechanical instability of silica-based films at lower baking temperatures. In any case, the refractive index of the silica hybrid is close to those of many polymers used for the fabrication of DBRs. Therefore, any effort to stabilize the Si–Hy upon treatment at lower temperatures would be meaningless. Figure 2a shows that the Si–Hy refractive index is slightly lower (*n* = 1.42 at 550 nm) than the one of both SiO_2_ and PAA. On the one hand, this can be symptomatic of the presence of partially reacted intermediates (indeed, the thermal analysis shows a weight loss and a reactive process at a higher temperature than the one used for the baking), and on the other hand, it can be due to the presence of porosity in the film.

Indeed, the porosity of the films was confirmed by AFM measurements (Figure S8), which also testify to the surface quality of the two materials that present a roughness of 0.2 and 0.3 nm for Ti–HY and Si–Hy, respectively. In both cases, the presence of porosity, coupled to small surface roughness, has the potentiality to make the materials suitable for sensing and photocatalytic processes, which require molecular species to intercalate within the DBR crystal (see below).

The extinction coefficient spectra of the two materials reported in Figure 2b agree with the observations done so far. While the silica hybrid is fully transparent in the entire spectral range analyzed, the titania hybrid function shows an absorption tail at 350 nm, which approaches a maximum at about 270 nm. The lower wavelength peak observed in *k* with respect to *n* is in full agreement with the theory of optical dispersion causal relation.^49^ No signature of light scattering is detected in the dispersion, indicating that the synthetic deposition process avoids drawbacks limiting the transparency of the films often observed in similar materials.^50^ Both materials are indeed fully transparent in the visible and near-infrared spectral ranges proving themselves promising for the fabrication of multilayered photonic structures.

### Multilayered Hybrid PhCs

**Figure 3:**
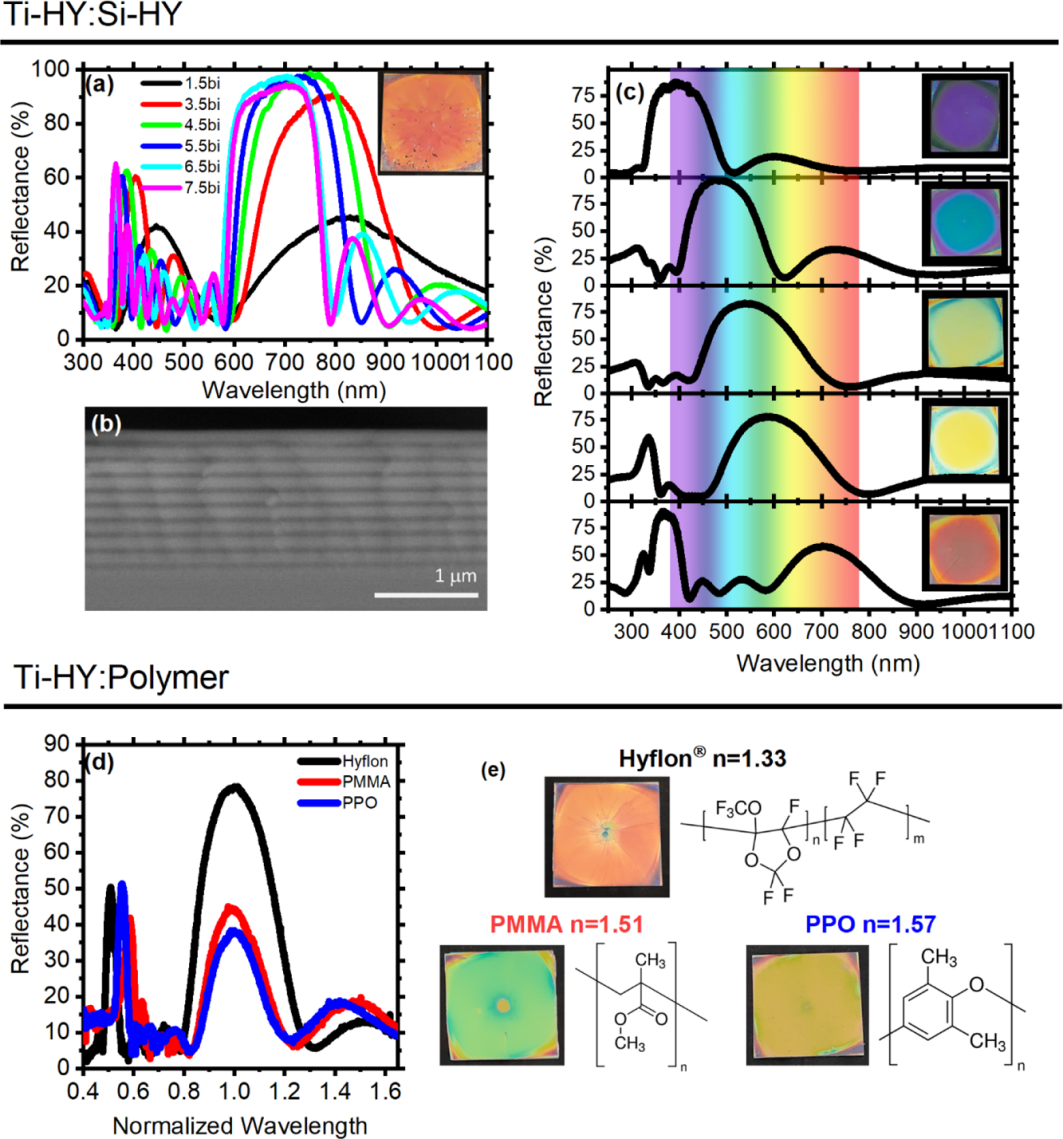
(a) Reflectance spectra for a Ti–Hy 97% v/v/Si–Hy 97% v/v multilayer collected after the deposition of 1.5, 3.5, 4.5, 5.5, 6.5, and 7.5 bilayers; the inset displays a digital photograph of each sample. (b) Cross-sectional SEM micrograph of the multilayer. (c) Reflectance spectra and digital photographs of DBRs with stop-bands tuned in different spectral ranges. Reflectance spectra (d) and digital photographs (e) collected for multilayers fabricated by alternating thin films of Ti–Hy with (from left to right) Hyflon AD 60 (black line), PMMA (red line), and PPO (blue line), see full characterization in Supporting Information Figure S13.

Once characterized, the hybrid materials were tested for the fabrication of multilayers, repeating subsequent alternating deposition of the two hybrids (Figure 1). Figure 3a shows the reflectance spectra of a Ti–Hy/Si–Hy structure for an increasing number of bilayers (bi). In this case, both the materials have been annealed at 300 °C for 60 s after deposition and contain a 97% v/v nominal concentration of inorganic oxide. The stop-bands are clearly detectable in the structure spectrum even with only three layers assembled (1.5 bi, black line) as two broad maxima centered at 830 nm and 444 nm are detected, corresponding to a first- and a second-order stop-band, respectively. On increasing the number of bilayers, these two features increase in intensity and shift toward the blue side of the spectrum. For samples made of more than 3.5 bilayers, the reflectance peak values approach 100%. In addition, the background of the spectra is dominated by interference fringes that arise from partial reflectance of the top and bottom DBR interfaces, indicating the good optical quality of the sample and lack of light scattering effects. The polarized angular dispersion of the spectral response certifies the optical quality of the structure and agrees with theoretical expectations,^1^ confirming the stop-band dispersion and thus the performance of the photonic structure (Figure S9). The homogeneity and flatness of layer thicknesses and sample surface is evident in Figure 3b,c, showing the cross-sectional SEM micrograph of a 9.5 bilayer sample cast on silicon and the digital photographs of the samples, respectively. In the SEM images, the Ti–Hy layers are visible in lighter tones while those made of Si–Hy are in darker tones and are homogeneous through the sample thickness. The digital photographs show DBRs with stop-bands tuned in the overall visible spectral region obtained by simply modifying the spin-coating conditions. Figure 3c shows five hybrid DBR samples made of 3.5 bilayers having stop-bands (from top to bottom) tuned at 400, 480, 540, 590, and 700 nm, respectively. The last two also show a second-order PBG at 340 and 370 nm, respectively. Notice that the intensity of the stop-bands in these samples increases gradually moving from the long to the short wavelength side of the spectrum, in agreement with the strong Ti–Hy refractive index dispersion (Figure 2b) that induces an increase of the DBR dielectric contrast upon decreasing the spectral position of the stop-band. In agreement with the spectral response, the digital photographs of the samples show bright color spanning from violet to red (insets in Figure 3c). The photographs show some color inhomogeneities at the edge of the substrates. This is typical of samples processed by dynamic spin-coating, where the edges of the squared substrate affect the spreading of the solution during its deposition.^1,50,51^ This is indeed commonly observed where square-shaped substrates^1,50,51^ are employed and can be reduced using round-shaped substrates.^52^

These results prove that the simple processing allows the controlled fabrication of photonic structures with very good optical quality with dielectric contrast as large as Δ*n* = 0.64 at 550 nm. Moreover, the porosity of the structures is promising for applications in sensing and photocatalysis, where analytes or reactive species need to intercalate within the lattice.

The high refractive index Ti–Hy was also tested in coupling with different polymer materials to fabricate both DBRs and microcavities. In this case, the hybrid was annealed at 80 °C only to avoid bringing the polymers above their glass-transition temperature as they would collapse under the weight of the dense Ti–Hy films. Notice that in this case, the Ti–Hy refractive index only approaches 1.89 at 550 nm. Demonstrating the possibility of treating the hybrids at low temperatures, but maintaining high dielectric contrast and coupling them with polymer matrices, makes them very interesting in light control applications. Indeed, while the purely hybrid structures show larger dielectric contrast, their processing together with several emitters would be hardly achievable on account of the relatively high annealing temperature needed for the Si–Hy films.^1^

Figure 3d,e shows the spectra and the digital photograph of three DBR samples made of alternating the perfluorinated low-index Hyflon AD 50 (*n* = 1.33), PMMA (*n* = 1.51), and poly(*p*-phenylene oxide) (PPO, *n* = 2.58) layers to Ti–Hy (*n* = 1.89 for 80 °C annealing) ones, providing a dielectric contrast of 0.56, 0.38, and 0.31, respectively. These polymers were selected as low-index materials as they are widely used in the field for sensing.^12,52^ Notice that, even though the dielectric contrasts are by far smaller than the value reached with the fully hybrid structure, the lower contrast obtained here is comparable to the maximum one at the state of the art for commercial polymers,^1,41^ while the highest is comparable to the most performing ones reported in the literature for similar structures.^9,39^ In Figure 3d, the reflectance spectra of the three polymer–Ti–Hy DBRs are reported versus wavelength normalized with respect to that of the stop-band maximum intensity. As expected, increasing the dielectric contrast between the two media increases both the intensity and line width of the stop-band.^1^ Moreover, the bright and consistent colors of the samples shown in Figure 3e confirm the high quality of the PhCs. Polarized angle-resolved transmittance measurements for all the three samples were performed to further confirm the stop-band dispersion (Figures S10–S12). Finally, cross-sectional SEM micrographs of the DBRs of Ti–Hy 97% v/v paired with different low-index materials demonstrate high homogeneity of the samples and allowed the retrieval of layer thicknesses for the materials. Reflectance spectra were simulated via TMM^53^ using the average layer thicknesses obtained from SEM analyses, refractive indices measured for the hybrids, and literature values for the polymers (see Supporting Information Figure S3). Table S1 also compares the measured and estimated thicknesses, which are in good agreement. This demonstrates the proper control achievable in constructing the DBRs and the thorough characterization of the structures.

A recent review^41^ compares the refractive indexes and the dielectric contrast of solution-processed films and DBRs made of commercial polymers and engineered materials. So far, the largest values reported belong to the polyvinyl alcohol–titania molecular hybrid reported by Stingelin and co-workers.^9,39^ They could obtain hybrid films with refractive indexes as large as 2.1 upon high-temperature annealing.^39^ Conversely, DBRs were obtained with milder treatment achieving *n* = 1.83 for the hybrid. The latter was coupled to a perfluoropolymer (*n* = 1.30) providing a dielectric contrast of 0.53. The values for our Ti–Hy/Si–Hy DBR are 0.64 at 550 nm, while when treated at low temperature and coupled with Hyflon, Ti–Hy offers dielectric contrast as high as 0.54. The good control in constructing DBRs with high refractive index is promising for the fabrication of high-dielectric-contrast microcavity structures doped with polymer or organic emitters. These families of emitters are interesting due to the simplicity of tuning their photoluminescence properties by synthetic routes, the ease of processing, and the high-quality films achievable. Indeed, polymers have often been used also to implement organic molecules and nanocrystalline emitters into cavity layers to achieve emission reshaping, lasing, and modification of the emission properties^1^ as they facilitate obtaining structures with optical quality and therefore performance, otherwise hardly achievable.^1,45,54,55^ For this purpose, we fabricated a microcavity inserting a defect layer made of F8BT (see the Experimental Section) in a PMMA/Ti–Hy structure (Figure S14). As discussed in the Supporting Information, the microcavity shows optical behavior in agreement with theoretical predictions proving the full compatibility of the hybrid with both polymer dielectric materials and emitters.^1^

### Application Perspectives

The ease of deposition of the hybrid DBRs, together with the evidence of porosity in the thin films, makes them interesting for applications unfeasible for common bulky inorganic planar structures with comparable dielectric contrast. To this purpose, we tested hybrid multilayers in photocatalytic and sensing systems, exploiting the well-known photoactive properties of titania^56^ and the photonic band structure to enhance light harvesting.

#### Harvesting Enhancement for Water Remediation

When a photoactive medium is integrated into a PhC structure, light absorption can be amplified within the medium itself at the stop-band edges owing to (i) light confinement and (ii) generation of slow photons propagating with reduced group velocity and thus with a longer lifetime and stronger interaction with the medium.^57−60^ In general, these effects are present for all wavelengths, even outside the stop-band, but enhanced at its edges.^58,60^ This approach has been widely demonstrated for opals^57^ that, on the other hand, can be fabricated only over small areas.^57,61^ Only few reports instead show the use of DBRs where the red edge of the stop-band is tuned on the absorbance of the dye instead of focusing on the photoactive medium.^58^ Therefore, integrating a photoactive medium in simple solution-processed porous PhCs is an interesting objective.

**Figure 4:**
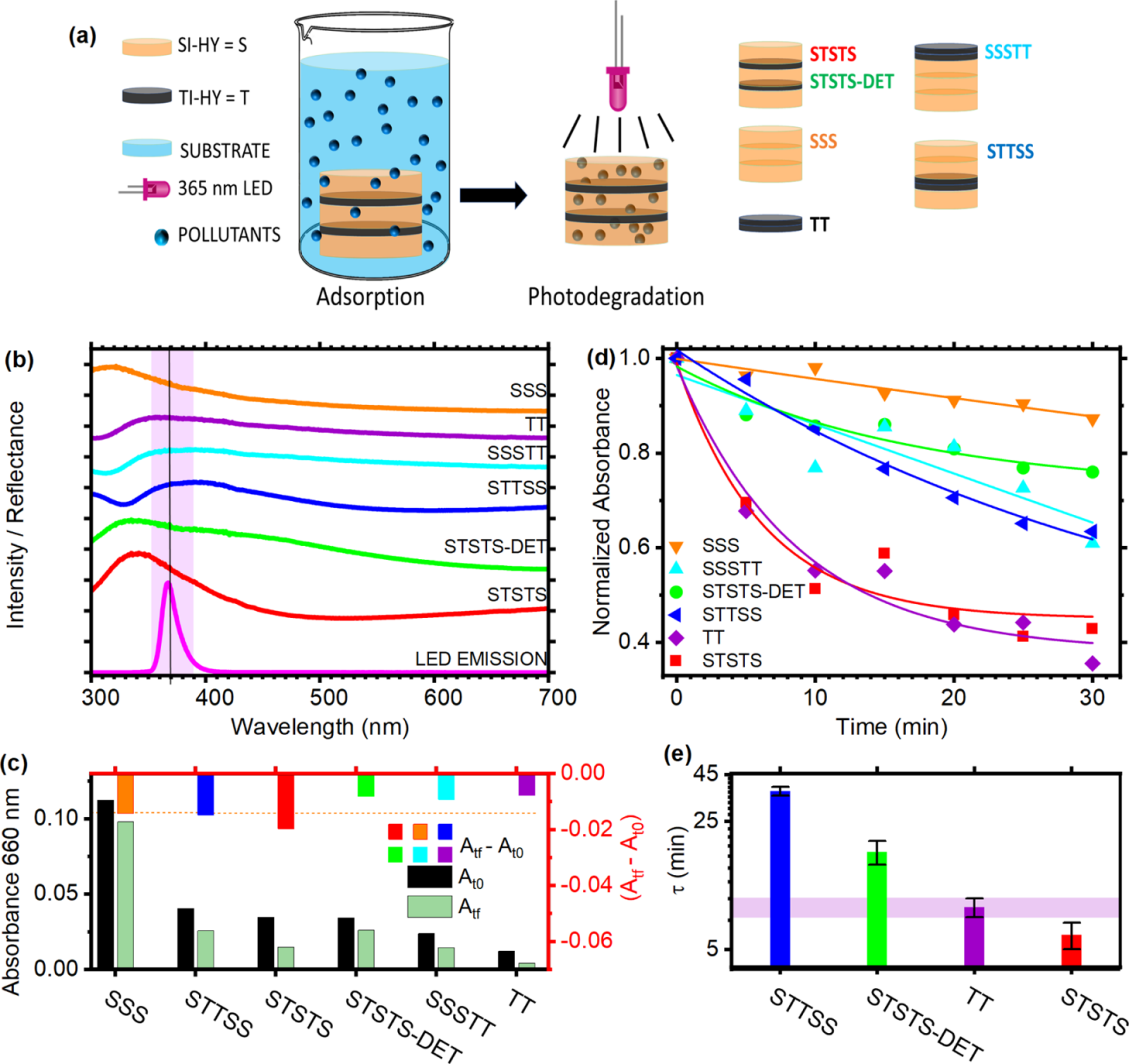
(a) Schematic of the photodegradation process of methylene blue (left side) and of the sample and references tested (right side). (b) Light source emission spectrum (pink) and reflectance spectra of all the samples and references investigated. (c) Left axis: dye absorbance at 660 nm wavelength before (black) and after 30 min of irradiation with UV light (green); right axis: difference between the two values. (d) Dye absorbance value in the multilayers along with the irradiation time (scattered data) and single exponential decay fits (lines). (e) Characteristic times τ retrieved from fitting the absorbance data, notice the log scale.

To demonstrate the suitability of the new structures as photocatalysts, we performed proof-of-concept experiments exploiting the well-known photocatalytic properties of titania.^62^ As illustrated in Figure 4a, DBRs and multilayers with different geometries were first immersed in a water solution containing 6 ppm of methylene blue at room temperature for 30 min and then exposed to a 365 nm LED light source (Figure 4b, purple line) while measuring the dye absorbance within the multilayers at set time intervals. A large number of reference samples were fabricated to assess the effect of the structure on both sorption of the dye and photocatalytic performances.

We fabricated one multilayer structure made of 2.5 bilayers of Ti–Hy/Si–Hy having the red edge of the stop-band spectrally superimposed to the emission source (STSTS in Figure 4b, red line), where enhancement effects should be maximized as the light emitted at such edge is confined into the high refractive index material.^57^ The structures were designed with 2.5 bilayers, and therefore only two layers of Ti–Hy, to demonstrate the process employing the minimum amount of the photoactive material, which is highly desired to minimize the costs and energy consumption in any perspective application. Nevertheless, having a larger number of periods could enhance the performances of the system. A second DBR (STSTS-DET) has thicker layers and thus stop-band shifted to the long wavelength side of the spectrum with respect to the first one (Figure 4b, green line). Having a larger amount of material, this structure should be more efficient in both the sorption process and the dye degradation, although the detuning of the stop-band with respect to the first sample should in principle decrease light absorbance and dye degradation efficiency. Other reference samples were fabricated for comparison:

(i) Two samples under the same conditions of the first sample (the same amount of material) but with layers arranged in a different order (SSTTS and SSSTT, Figure 4a blue and cyan line). These two samples should guarantee similar dye sorption with respect to the first sample, but lower degradation efficiency as the stop-band is not present in the structures.
(ii) Two references made of three layers of Si–Hy (SSS, orange line) and two layers of Ti–Hy (TT, black line), respectively. The first serves to assess the dye degradation occurring upon UV light irradiation without photoactive medium, while the second serves to assess the effect and the photoactivity of Ti-Hy by itself.

The reflectance spectra of all samples are reported in Figure 4b as compared to the LED emission spectrum (351–407 nm), superimposing the Ti–Hy absorbance onset (see Figure 2b). All dye absorbance spectra collected along the UV exposure are reported in Figure S14. We evaluated three figures of merit: (i) the amount of dye intercalated within the structure, (ii) the amount of dye degraded after UV illumination, and (iii) the kinetics of degradation. The first was assessed by evaluating the absorbance of the dye within the structure at λ = 660 nm (see Figure S15), which in agreement with the Lamber–Beer law has a linear dependence on the concentration of the dye. The data are reported in Figure 4c (black bar) and show that the sample bearing a larger dye concentration after the sorption process is the one made only of silica hybrid (SSS), while the titania one (TT) show the smallest value. These data, in agreement with AFM characterization, suggest that while the silica shows larger porosity, the titania layers are more compact and, in principle, can even hinder the dye diffusion within the multilayers. Indeed, the sample having two titania layers on top of the structure (SSSTT) shows the second-to-last concentration of dye, while the values for the other samples are comparable and show that the multilayered structure induces a 3.5-fold increase of the dye intake with respect to the bare TT sample. The dye concentration in the sample after 30 min of irradiation is reported in Figure 4c as a green bar, the difference between the two values as (*A*_*t*30_ – *A*_*t*0_) is reported on the left *y*-axis of the same plot (top bars with same colors as Figure 4b). Among all the samples we observe, the bare Ti–Hy shows the smallest value, confirming that lower porosity causes lower dye intake. The pure Si–Hy sample instead shows a remarkable degradation since the dye itself is not very stable under UV irradiation. All the other references show values in-between these two samples, suggesting that the process is limited by the diffusion of the dye during the sorption step. Remarkably, despite bearing a relatively low amount of dye, the sample with the red edge of the stop-band tuned to the LED emission peak shows the largest degradation values with a 2.5-fold enhancement with respect to the TT layer, suggesting that photonic effects occur in this sample. This is also backed up by the degradation kinetics.

Figure 4b shows the absorbance value at 660 nm for all the samples normalized by the value measured before the exposure (*A*_*t*0_), which also represent the fraction of degraded dye. In this respect, the fraction of dye of the STSTS sample with respect to the initial concentration approaches the one of the TT sample. Nevertheless, the initial amount of dye in the DBR was about 10 times larger with respect to the TT reference, demonstrating that the multilayers enhance the overall degradation. The data were fit with a single exponential decay trend to retrieve a characteristic time of the process (τ), reported in Figure 4e (data not shown for the only SSS and for the SSSTT sample, which shows an almost linear and very slow decrease within the investigated timeframe with a characteristic time approaching 10^3^ s). While all the references have characteristic times larger than the bare Ti–Hy film (τ_TT_ = 8.5 ± 1 s), the sample STSTS (τ_STSTS_ = 6 ± 1 s) shows a decrease of 28 ± 20% with respect to the former, confirming the positive effect of the photonic structure on the process kinetics.

These preliminary data confirm that the Hy–Ti films are photoactive in the degradation of methylene blue and that it is possible to tune the dye intake operating on the geometry of the multilayers. Moreover, data also suggest that DBRs are promising structures for photon harvesting enhancement. When the red edge of the stop-band is tuned to the wavelength of the activating light for the photocatalytic process—through an accurate engineering of the geometry of the DBRs—it is indeed possible to obtain a 2.5-fold enhancement of dye degradation, which currently represent an advancement with respect to previously reported literature where highly porous inversed opals are employed (see refs (61) and (63) and references therein). The results are instead comparable with previously reported DBR structures^64^ that, on the other hand, report on the tuning of the stop-band on the dye intake, limiting the suitability of the structure only to molecules absorbing in the visible range of the spectrum.^58^

#### Sensing

The DBR porosity can in principle be exploited to assess molecular species in the liquid state intercalating within the DBR structure. The spectral position of a DBR stop-band depends indeed on its effective refractive index, as described by the Bragg–Snell law (eq S1).^1,65−67^ In turn, the effective refractive index of the single layers that compose the DBR depends on their components, which in our case are the Ti and Si hybrids and the air in the porosity and on their filling volume.^48^ In agreement with eqs S1–S6, filling the porosity with a liquid analyte increases the effective refractive index of the single layers, and in turn of the whole DBR, thus red-shifting the stop-band spectral position. In principle, this can be achieved for any analyte able to intercalate within the structure and then to increase its effective refractive index.^66^

**Figure 5:**
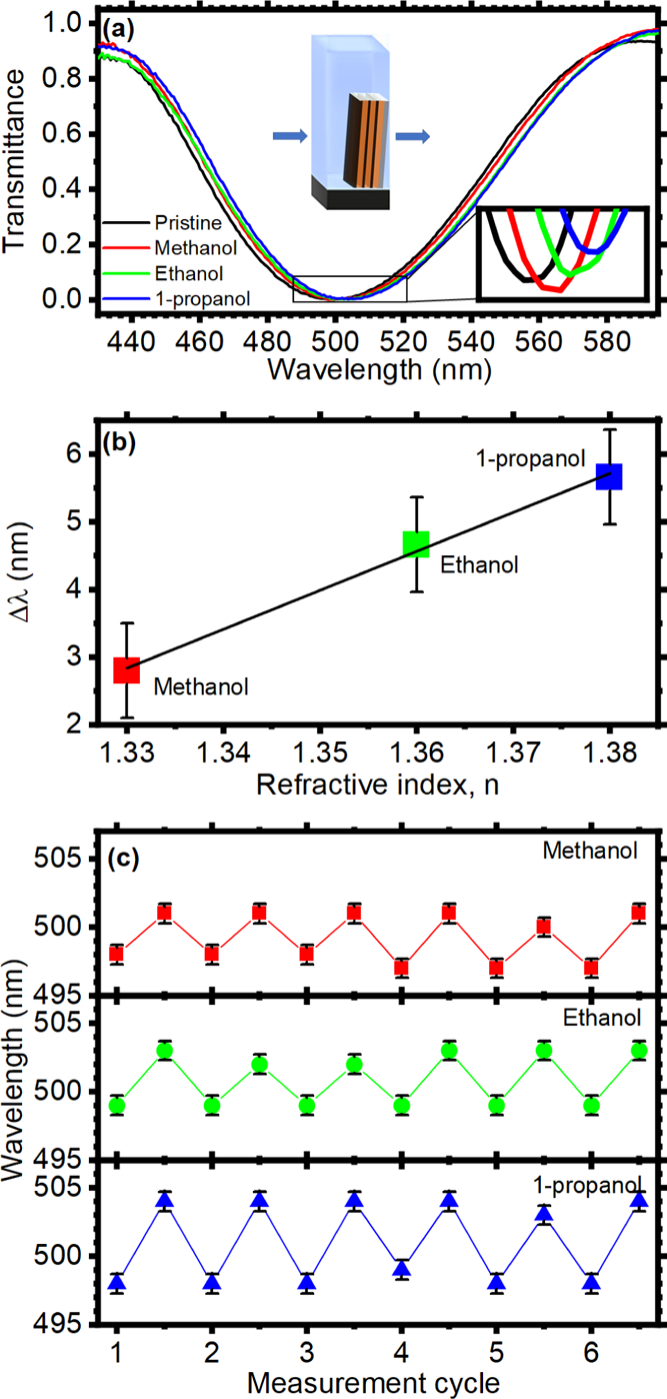
Optical response of a Ti–Hy 97% v/v/Si–Hy 97% v/v DBR made of 2.5 bilayers before (black) and after exposure to methanol (red), ethanol (green), and 1-propanol (blue) solution. (a) Transmittance spectra and the insets show a magnification of the curves and the schematic of the measurement setup. (b) Spectral shift as a function of the analyte refractive index. (c) Spectral position of the stop-band of the same DBR before and after exposure for seven cycles to the three analytes.

To demonstrate the possibility of using the hybrid structures as optical sensors, we exposed a 2.5 bilayers DBR to methanol (*n* = 1.33), ethanol (*n* = 1.36), and 1-propanol (*n* = 1.38) in the liquid phase while measuring the sample transmittance, as sketched in Figure 5a. Alcohols were chosen as proof-of-concept analytes due to chemical similarity among compounds with small refractive index differences. The transmittance spectrum of the pristine DBR shows a minimum positioned at 498 nm (*n*_air_ ∼ 1) that shifts to longer wavelengths after the analyte intercalations, as highlighted in the inset of Figure 5a,b reporting the spectral shift of the stop-band transmittance minimum (Δλ) over the analyte refractive indexes. In agreement with the small effective refractive index variations and with the literature^1,65−67^ (see also eqs S1–S6), the shift value increases linearly with the refractive index of the analytes. Notice that due to the spectral resolution of the instrument employed (1.4 nm), the structure allows easily distinguishing methanol from propanol and ethanol, although the shift obtained for ethanol and 1-propanol is within the error bar of the measurement. The system allows indeed a lower detection limit of 0.024 refractive index units (RIU) and provides a shift of 57 nm per RIU.

We also investigated the reversibility of the sensor response. Figure 5c shows the spectral position of the stop-band peak before and after seven cycles of exposure and desorption at room conditions for the three analytes, noting that all the measurements for all analytes were performed with the very same sample. The data show consistency along all the seven cycles for all the analytes, confirming the reversibility and the reproducibility of the measurement. As the results obtained are comparable with many reported for porous structures fabricated implementing the high-temperature calcination process,^66^ complex deposition procedures,^34^ or layer-by-layer techniques,^67^ the ease of processing of the proposed structure makes them very interesting for sensing applications.

## Conclusions

In conclusion, we reported the synthesis of stable polymer–inorganic hybrid thin films and DBRs with tunable refractive indexes via an easy sol–gel processing that does not require furnace annealing and does not imply the formation of strong acidic species. Coupling hybrids fabricated from PAA and alkoxides of silicon or titanium allows achieving a dielectric contrast of Δ*n* = 0.64 at 550 nm, which to the best of our knowledge is the highest reported for planar solution-processed DBRs. Moreover, the proposed method simply exploits thermal annealing on a hot plate and can be run at low temperature. This favors stable Ti–Hy thin films which can be coupled with several polymers for the fabrication of DBRs and microcavities doped with polymer emitters. This compatibility is also possible thanks to the stability of the Ti–Hy, which is not soluble in organic solvents, and to the mild sol–gel conditions employed, which do not affect polymer dielectrics and emitters. These findings, together with a maximum dielectric contrast obtained for polymer/Ti–Hy DBRs (Δ*n* = 0.54), show promising applications often forbidden to solution-processed polymer structures owing to their intrinsically low dielectric contrast. As a proof of principle, thanks to layer porosity, hybrid DBRs were used for refractive index detection and for the enhancement of light harvesting and pollutant intake in the photocatalytic degradation process, where Ti–Hy acts as the photoactive medium. In detail, we investigated the possibility to detect small refractive index variations in liquid media with similar chemical properties achieving a lower detection limit of 0.024 RIU and a stop-band shift of 57 nm/RIU. As regard to photodegradation, the Ti–Hy photoactivity within the multilayered structure was demonstrated through the degradation of methylene blue under UV irradiation, while a detailed analysis of the dye degradation kinetics allowed us to disclose the role of pollutant diffusion and the photonic structure in enhancing pollutant intakes and photon harvesting, respectively. These results pave the way to the development of materials that expand the arsenal of industrially scalable photonics.

## Supporting Information Available

Hybrid sol–gel mechanism and reaction products; digital photographs of sols; hybrids’ thermogravimetric and calorimetric analyses; reflectance spectra of thin films during thermal annealing; FT-IR analyses of the hybrids during thermal annealing; optical function of the hybrids: effect of annealing temperature, aging, and polymer content; AFM characterization of the hybrid surfaces; DBR angular-resolved transmittance spectra collected for P and S polarizations; SEM cross-sectional micrograph of full hybrids and polymer-hybrid structures with relative experimental and calculated spectra; full optical characterization of a hybrid polymer/Ti–Hy microcavity structure, methylene blue absorbance in the multilayered structures during the photodegradation process; and effect of refractive index variation in the DBR porosity (PDF)

## Abbreviations

AFM: atomic force microscopy
ATR: attenuated total reflection
bi: bilayer
DBR: distributed Bragg reflectors
DCS: differential scanning calorimetry
FT-IR: Fourier transform infrared spectroscopy
LED: light emitting diode
PAA: poly(acrylic acid)
PhCs: photonic crystals
PPO: poly(2,6-dimethyl-1,4-phenylene oxide)
SEM: scanning electron microscopy
Si–Hy: polymer–silica hybrid
TEOS: tetraethyl orthosilicate
TGA: thermogravimetric analysis
TIBU: titanium(IV) butoxide
Ti–Hy: polymer–titania hybrid
