# Supplementary material for: Mild Sol–Gel Conditions and High Dielectric Contrast: A Facile Processing toward Large-Scale Hybrid Photonic Crystals for Sensing and Photocatalysis
Source: ACS Appl Mater Interfaces. 2022 Apr 21;14(17):19806–17. doi: 10.1021/acsami.1c23653 (PMC9073830; doi:10.1021/acsami.1c23653)
Supplement: Supplementary file 1 — am1c23653_si_001.pdf [file am1c23653_si_001.pdf]

SUPPORTING INFORMATION

Mild sol-gel conditions and high dielectric contrast: a facile processing towards large-scale hybrid photonic crystals for sensing and photocatalysis

*Simone Bertucci,[1,2] Heba Megahd,[1] Andrea Dodero,[1] Sergio Fiorito,[2] Francesco Di Stasio,[2] Maddalena Patrini,[3] Davide Comoretto,[1] Paola Lova[1]**

[1] Dipartimento di Chimica e Chimica Industriale, Università degli Studi di Genova, Via Dodecaneso 31, 16145, Genova, Italy.

[2] Photonic Nanomaterials, Istituto Italiano di Tecnologia, Via Morego 30, 16163 Genova, Italy

[3] Dipartimento di Fisica, Università degli Studi di Pavia, Via A. Bassi 6, 27100, Pavia, Italy.

Corresponding Author

Paola Lova, E-mail: paola.lova@unige.it

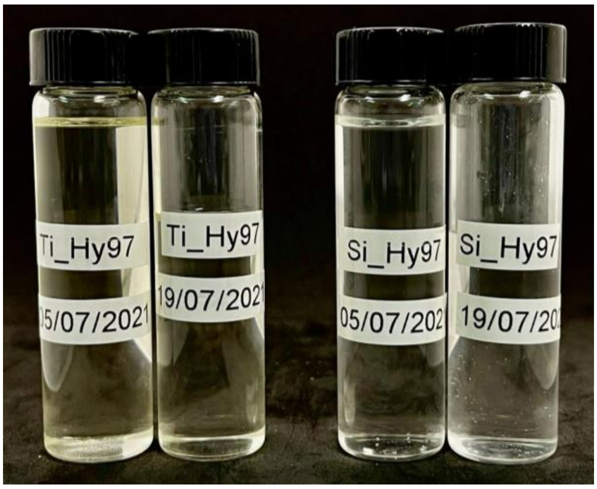

Figure S1. Digital photograph of titania (left) and silica (right) sols as prepared and after 2 weeks kept at room temperature.

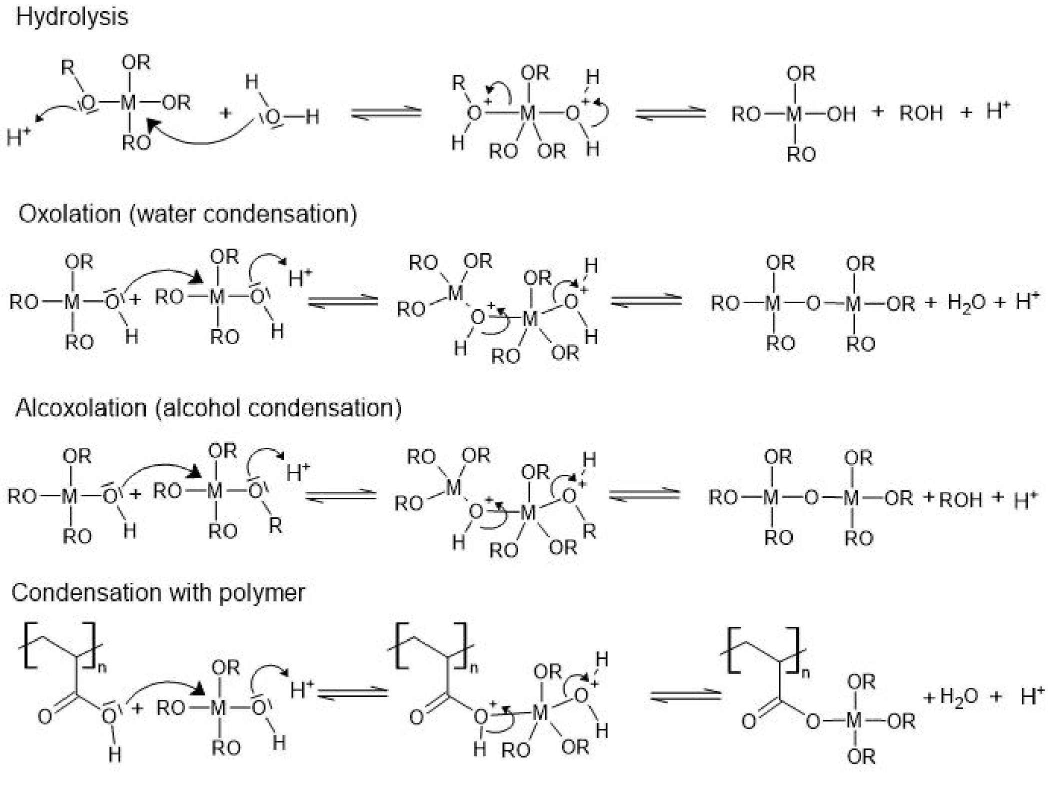

Scheme S1. Schematic of acid-catalyzed hydrolysis and condensation reactions including the condensation between the inorganic oligomers and the polymer.

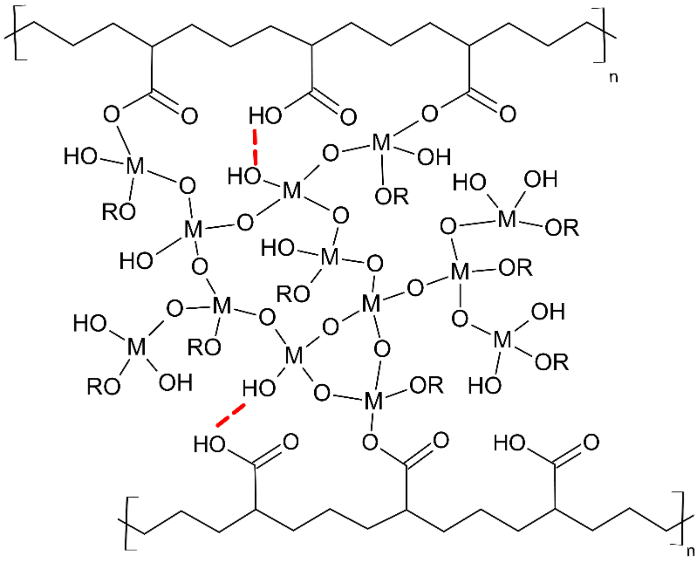

Scheme S2. Proposed chemical structure of the hybrids where the inorganic matrix interacts with the polymer via covalent (black continuous line) and hydrogen bonds (red dashed line).

DSC thermograms carried out on the titania-based xerogels show a broad endothermic peak in the range between 50 and 150 °C (Figure S1 a-c) assigned to the evaporation of butanol which has a boiling point of 118°C. The TGA reported in Figure S1b for the same samples confirms the hypothesis of evaporation of solvent, showing a gradual loss of weight in the same range of temperature. At about 300 °C a sharp endothermic peak can be seen in the DSC thermograms associated with a sudden weight loss in the TGA curves. As phase transitions are not usually accompanied by a loss of weight, the intense endothermic peak could be then assigned to a reactive event with the release of volatile secondary products. Last, a sharp exothermic peak at around 400°C, which is not related to any change in weight, is attributed to the crystallization of amorphous titania to a crystalline phase.[1] Remarkably, the characteristic temperatures of the 70% v/v Ti-Hy sample are slightly shifted to higher values with respect to the other two samples. Such a finding may be related to the greater content of PAA within the hybrid structure, which in turn plays a stabilizing effect and may cause a delay in the formation of TiO_2. The differences in weight loss observed between the samples are ascribable to the released of trapped solvent and condensation products (mainly water and alcohols) which increases increasing the inorganic content of the gel (see reaction Scheme S1). As a reactive process with release of volatile by-product occurs during the measurement, TGA and DSC data only allow to assess reaction temperature.

Concerning the silica hybrids, DSC results show two overlapping broad and rather weak peaks between 100 and 200 °C which can be assigned to the evaporation of butanol (T_b = 118 °C) and unreacted TEOS (T_b = 168 °C) respectively (Figure S1b). In the same range of temperatures weight losses are observed in TGA analysis (Figure S1d). The reason why the second peak results shifted to higher temperature for the higher inorganic content sample (97%, red line) compared to the lower one (92%, black line) might be due to slight differences in the extent of the hydrolysis and condensation reactions, which impact the quantity of unreacted precursor in the samples. An endothermic peak is visible at about 500 °C (at the upper-temperature limit of the instrument) in the DSC of Figure S1b. In the relative TGA thermogram a weight loss can be identified at the same temperature An endothermic peak accompanied by weight loss could suggest a similar reactive event like the one evaluated for the titania-based sols.

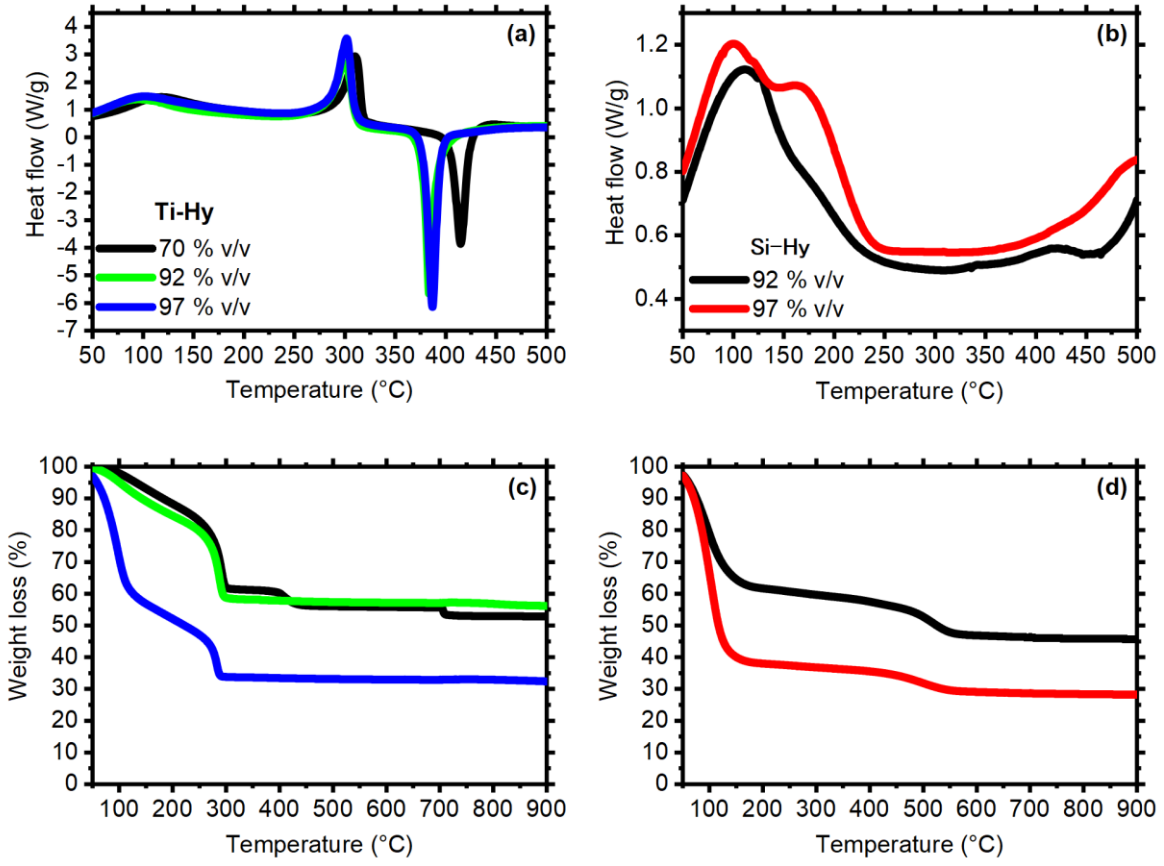

Figure S2. DSC (a, b) and TGA (c, d) data for the Ti-Hy (a, c) and Si-Hy (b, d) materials with different nominal contents of oxide.

The optimal annealing time for the films was assessed by measuring the UV-vis-NIR transmittance spectrum of a thin film during the thermal treatment using a heat-resistant reflectance probe (Supporting Information Figure S3 and S4). Reflectance spectra were acquired continuously over time and plotted as a contour plot where reflectance percentage is reported as color scale in a wavelength (x) - time (y) plane. Figure S3 shows data for titania-based hybrids while Figure S4 reports the results obtained for silica-based hybrids for different reaction times. The spectra reported in panels a and c show the formation of interference fringes upon annealing. The contour plots are indeed characterized by the formation of an interference pattern after few seconds of annealing testifying the generation of a plane parallel thin film. Maxima and minima of reflectance are assigned to an interference pattern that shifts towards shorter wavelengths during the first one minute of annealing and then remains constant for both samples. This effect suggests that after one minute of thermal treatment the titania hybrids reach a steady-state condition which implies that further heating of the samples does not cause any additional effect. In Figure S4, data for silica hybrids show similar trends compared to the ones for titania hybrids. In this case, during the first 60 seconds of thermal treatment. the samples never reach a steady-state condition as the spectral features do not cease to shift throughout the annealing process. On the other hand, the variation is rather weak compared to the one observed for the titania hybrid films. The continuous variation throughout the treatment confirms what DSC thermograms suggested, that is the reactive process could need higher temperatures to be completed.

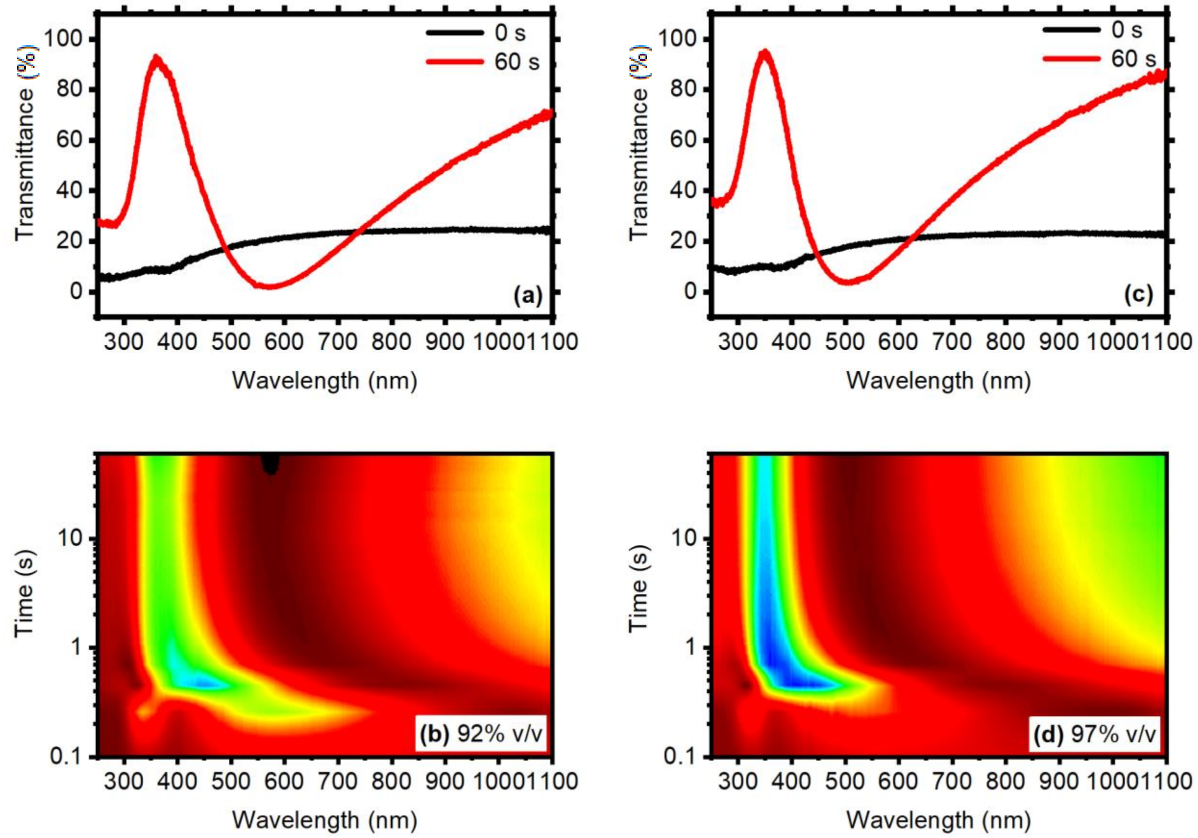

Figure S3. (a, c) Transmittance spectra collected before and after 60 second of annealing and (b, d) contour plots of the transmittance spectra measured while annealing films of Ti-Hy at 300 ° for (a, b) 92% v/v and (c, d) the 97% v/v nominal TiO_2 concentration.

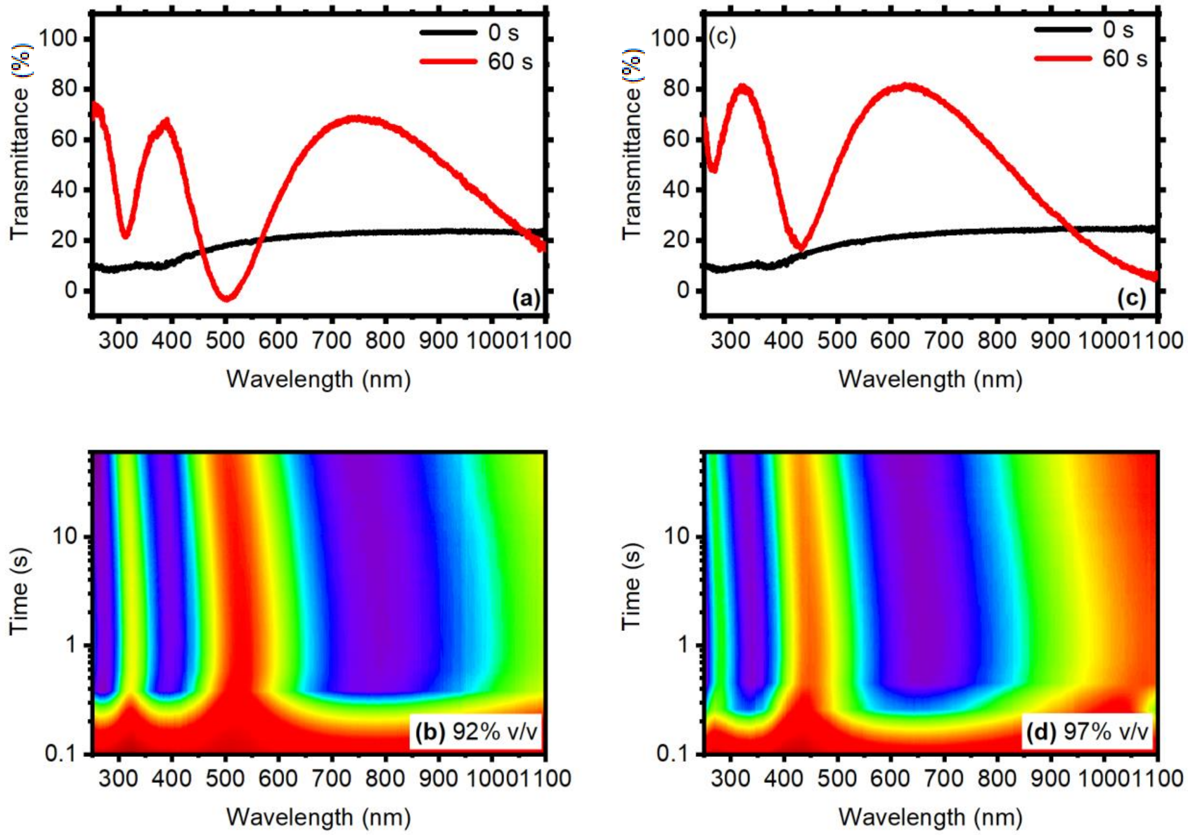

Figure S4. (a, c) Transmittance spectra collected before and after 60 s annealing and (b, d) contour plots of the transmittance spectra measured while annealing at 300°C for films of Si-Hy obtained by spin-casting for the 92% v/v (a, b) and 97% v/v (c, d) nominal SiO_2 concentration.

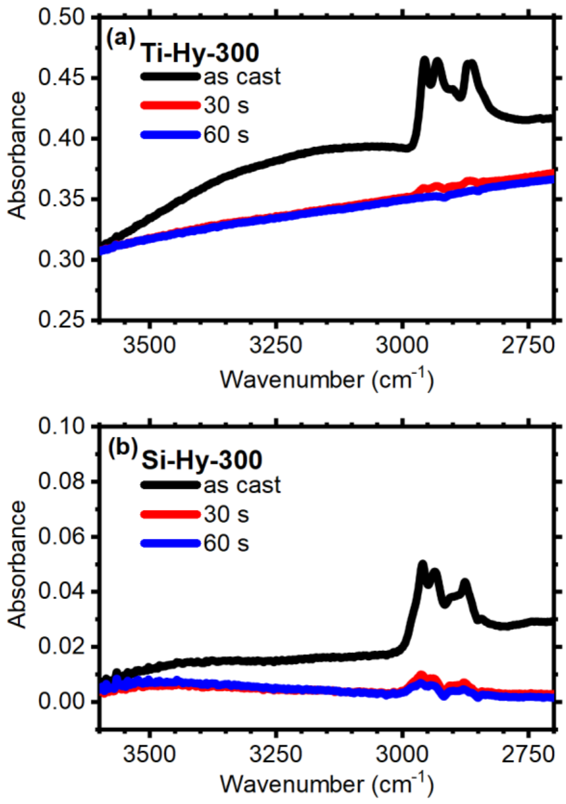

Figure S5. FTIR ATR spectra collected for the two hybrids (97% v/v) before and after thermal annealing at 300°C for 30 (red line) and 60 s (blue line). (a) TiO_2-Hy; (b) SiO_2-Hy.

Figures S6 shows the dispersion spectra of refractive index for Ti-Hy annealed at 80 °C and 300 °C after casting and after 10 days at room conditions. The index values remain substantially unchanged aside from a minor increase of the values in the entire spectral range, probably due to gradual conversion of remaining traces of unreacted precursor.

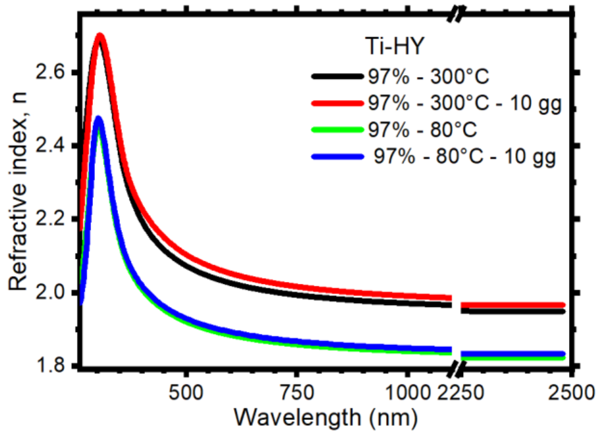

Figure S6: Refractive index spectra of the Ti-Hy annealed at 80°C and 300°C as cast and after 10 days at room conditions.

Figure S7 reports the dispersion of refractive index for fully inorganic titania films found in the literature (TiO_2)[2-3] and compares it with the values obtained for hybrids with a different nominal concentration of inorganics. The results for Ti-Hy with 70% v/v (green line), 92% v/v (orange line) and 97% v/v (red line) show lower values than the pure oxides in agreement with the presence of an increasing amount of polymer content. The results obtained for Ti-Hy 92% and Ti-Hy 97% are indiscernible and slightly lower than the values found in the literature for fully inorganic titania films. The results for Ti-Hy 70% are lower than the other hybrid compositions, in agreement with the larger PAA content. Similarly, the two Si-Hy casts with 92% v/v and 97% v/v inorganic load are superimposable.

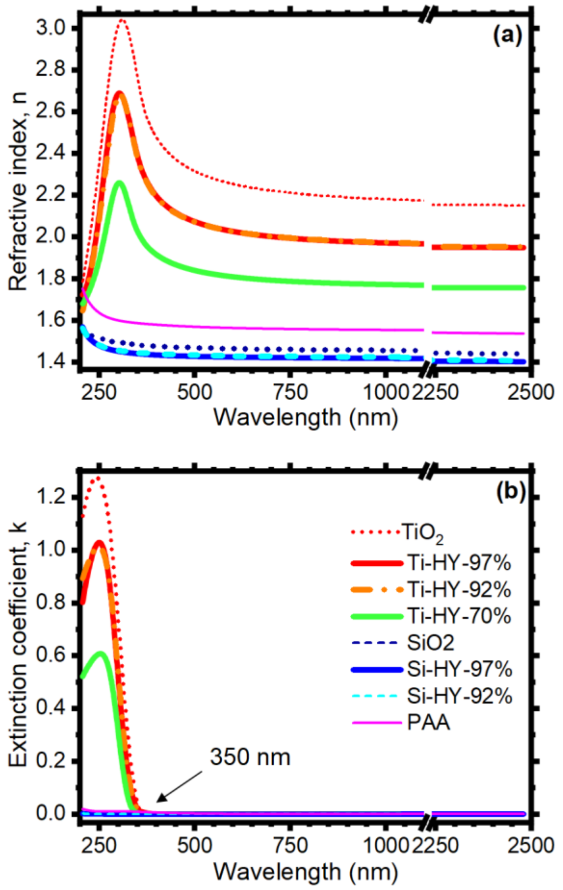

Figure S7. Refractive index (a) and extinction coefficient (b) dispersion retrieved for thin films of Ti-Hy annealed at 300°C and bearing different nominal TiO_2 content (97% v/v in red, 92% v/v in orange, 70% v/v in green) and of Si-Hy annealed at 300°C (97% v/v in blue, 92% v/v in cyan) compared to the spectra of pristine compact silica and titania as retrieved from the literature,[2-3] and of PAA.

Figure S8 displays the AFM images collected for the different hybrids. The profile data indicate that when the Ti-Hy is annealed at 300°C a surface roughness as low as 0.2 nm is obtained. The values are slightly larger for the Ti-Hy annealed at 80°C (0.3) nm and for the silica hybrid (0.2-0.3 nm). Interestingly, all samples show a particle-like surface and not a compact material. These data suggest the voids within the surface that could be ascribed to porosity.

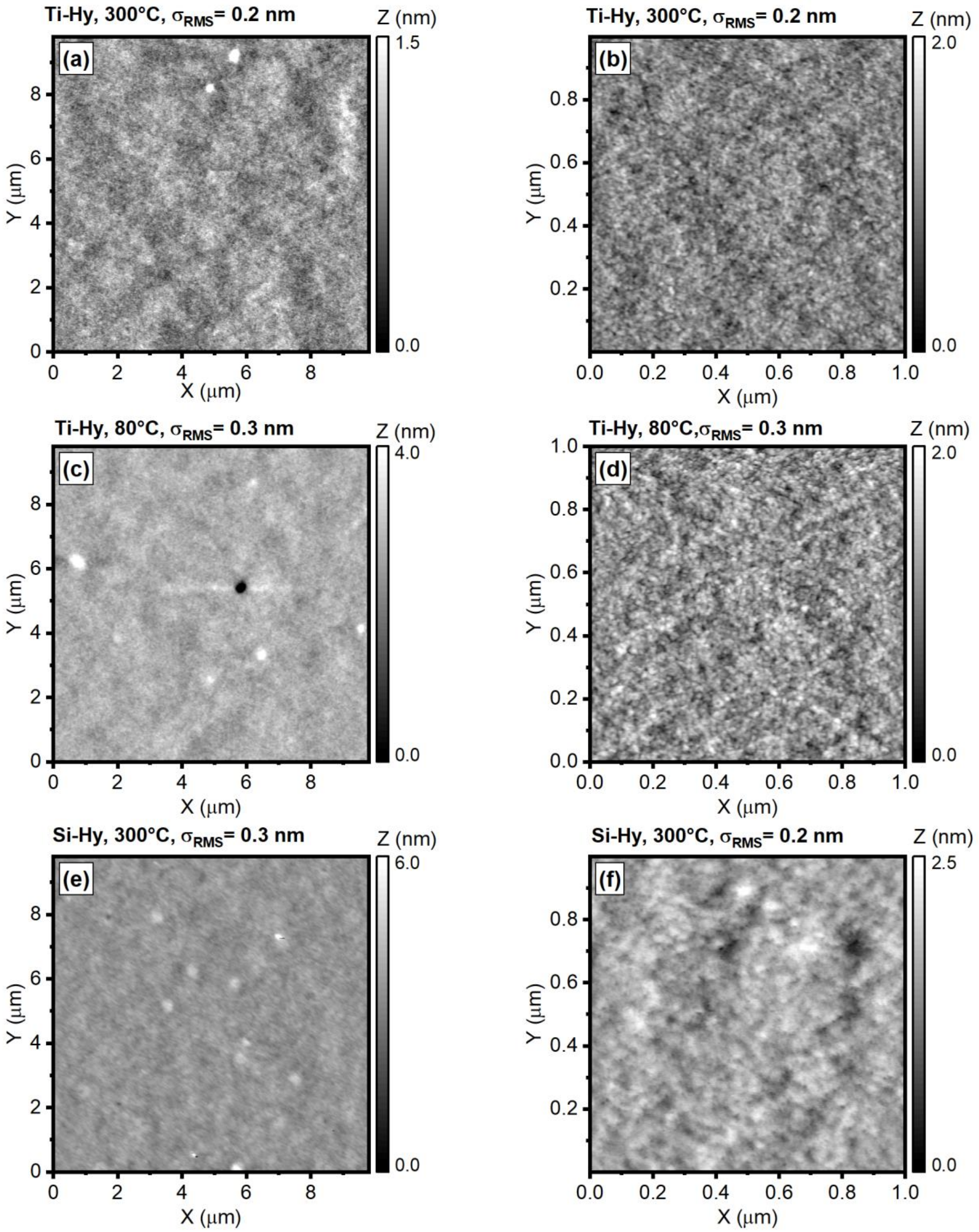

Figure S8. AFM micrographs for Ti-Hy 97% v/v annealed at 300°C (a,b) and at 80°C (c,d) and for Si-Hy 97% v/v annealed at 300°C (e,f).

Figures S9, S10, S11, and S12 show the angle-resolved transmittance spectra of the DBRs reported in Figure 2 (see comments in the main text). For all spectra we notice a blue shift to the short wavelength side of the spectrum with increasing collection angles. For s-polarization the stop-band width remains substantially unchanged, while for p-polarization both width and intensity decrease approaching the Brewster angle for the respective structure in full agreement with theoretical predictions.[4]

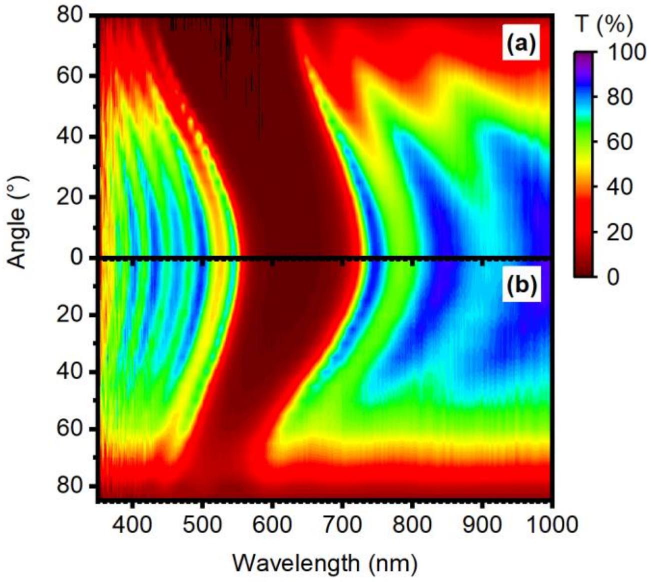

Figure S9. Angle-resolved transmittance spectra for a Si-Hy:Ti-Hy multilayer made of 7.5 layers for s (a) and p (b) light polarization.

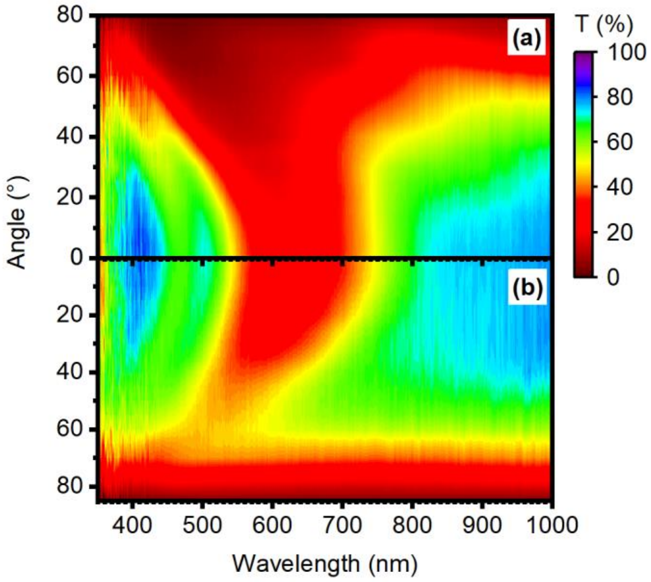

Figure S10. Angle-resolved transmittance spectra for a Ti-Hy: Hyflon®multilayer made of 4.5 layers for s (a) and p (b) light polarization.

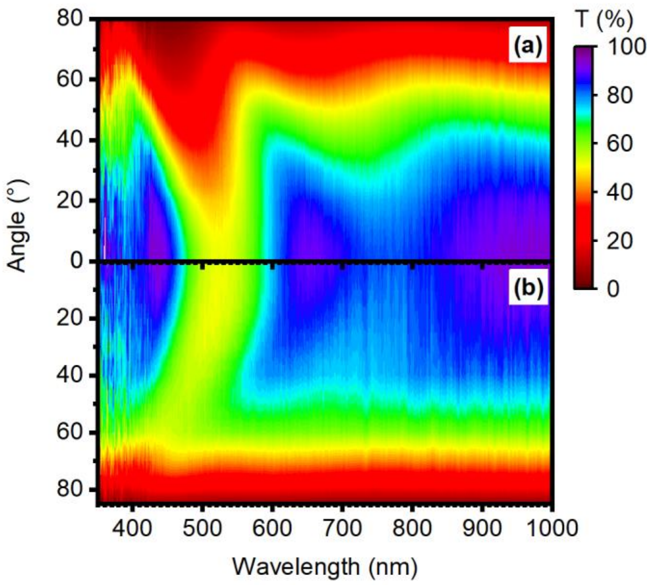

Figure S11. Angle-resolved transmittance spectra for a Ti-Hy:PMMA multilayer made of 3.5 layers for s (a) and p (b) light polarization.

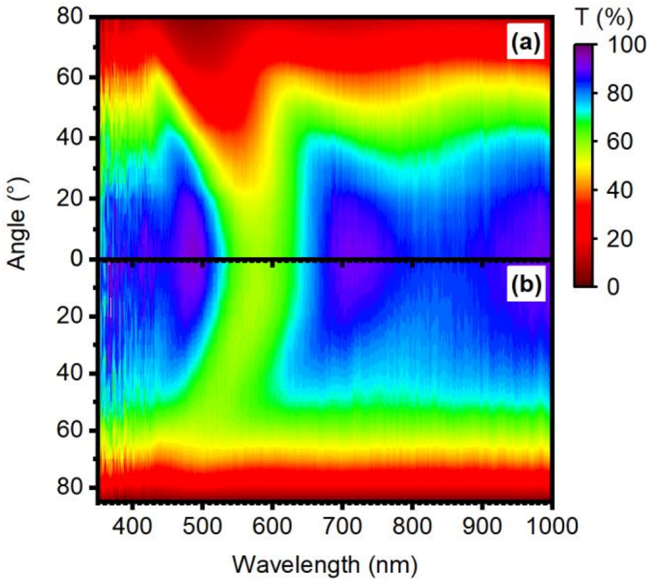

Figure S12. Angle-resolved transmittance spectra for a Ti-Hy:PPO multilayer made of 3.5 layers for s (a) and p (b) light polarization.

Figure S13 reports the calculated and experimental spectra (a1-d1) and SEM micrographs (a2-d2) for DBRs made of Hy-Ti (97% v/v) coupled to Hy-Si (97% v/v), PMMA, PPO and Hyflon.

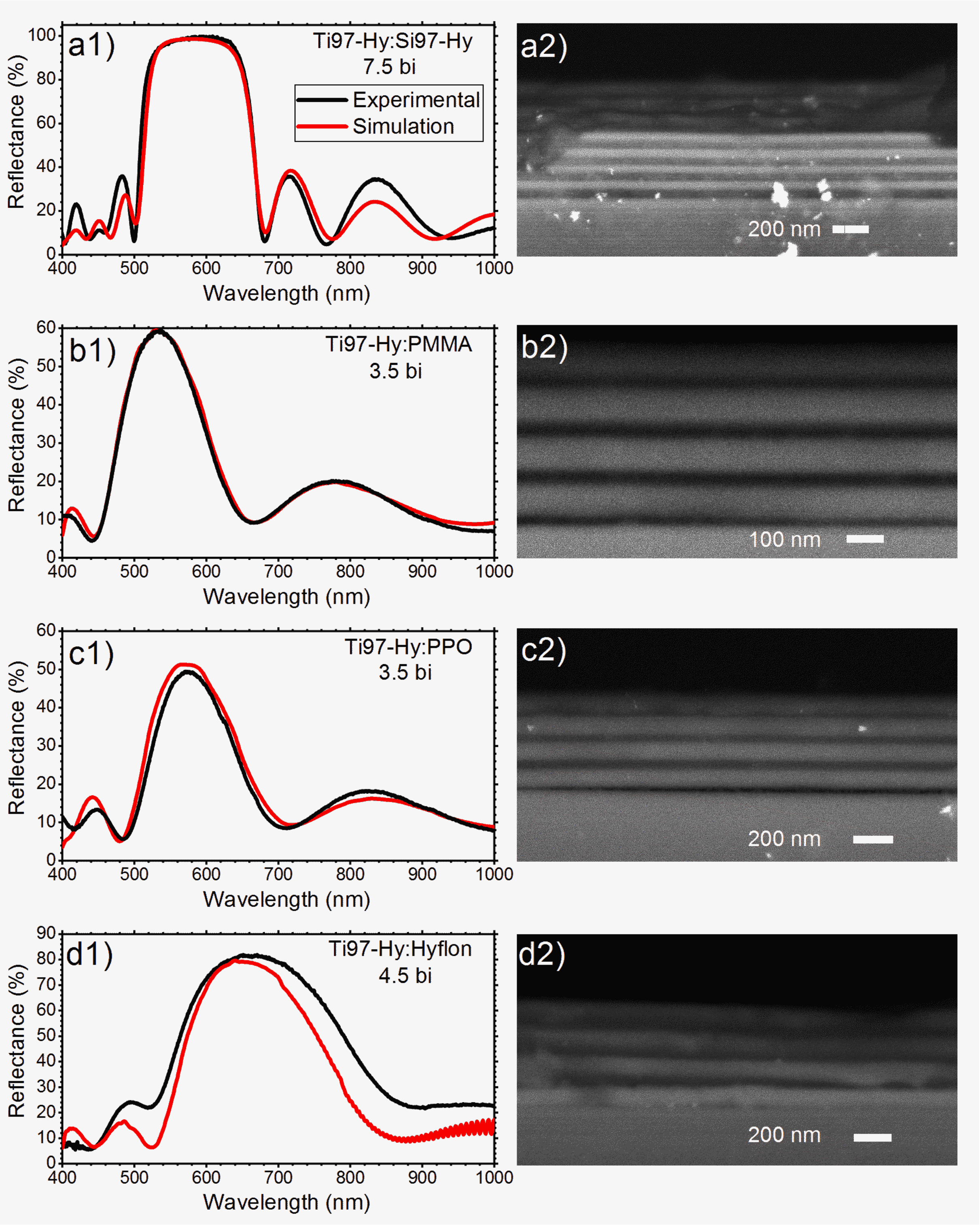

Figure S13: a1-d1) Experimental (black) and simulated (red) reflectance spectra of DBRs and a2-d2) the corresponding SEM micrograph for samples a) Hy-Ti:Hy-Si, b) Hy-Ti:PMMA, c) Hy-Ti:PPO, Hy-Ti:Hyflon. Hybrid inorganic concentration is (97% v/v).

In the SEM, the lighter layers represent the Hy-Ti while the darker ones represent the low-index materials. The average thicknesses extracted from multiple SEM micrographs for the low and high index materials are reported in Table S1. As the sample preparation for SEM involves a freeze-dry cracking after cooling the sample in liquid nitrogen, fine debris and inhomogeneous cracking are seen in the micrographs. However, good homogeneity is still observed for most samples. The experimental spectra collected for the samples were then modelled using the thicknesses retrieved and the materials refractive indexes. For this purpose we used the measured indexes for Hy-Ti (97 % v/v) treated at 80°C and 300°C and of Hy-Si (97 % v/v) and values retrieved from literature for PMMA[5], PPO[6] and Hyflon® (from Solvay Specialty Polymer S.p.A, Hyflon® PFA Design & Processing Guide). For the calculation, the average thickness values obtained from the SEM measurements were used as an initial input and adjusted slightly to obtain a good fit to the measured reflectance. Tables S1 show the good agreement between the layer thickness used in the simulations with the measured ones. For the DBRs made of Ti-Hy alternated to Si-Hy, PMMA, and PPO, the simulations show great agreement with the experimental data in terms of spectral position, intensity, and width of both stop-bands and interference fringes. The simulated spectrum for the Ti_Hy:Hyflon® DBR show minor differences attributed to a slightly larger variation between the layer thicknesses, that can clearly be seen in the SEM images compared to other samples.

Table S1: Average layer thickness value obtained from SEM measurements and those used in simulations. All hybrids have concentration 97% v/v.

Sample	N° bilayers	d_H SEM (nm)	d_H Simulation (nm)	d_L SEM (nm)	d_L Simulation (nm)
**Ti-Hy:Si-Hy**	4.5	79±8	86	74±15	80
**Ti-Hy:PMMA**	3.5	97±5	97	34±6	47
**Ti-Hy:PPO**	3.5	93±7	95	46±9	57
**Ti-Hy:Hyflon**	7.5	90±25	92	40±8	57

Figure S14 reports the structure (a) and the reflectance spectrum (b, red continuous line) of a microcavity casted alternating layers of PMMA (n=1.51) and Ti-Hy (n=1.89, baking at 80 °C) to form two DBRs made of 14.5 periods and sandwiching a layer of F8BT in between. The spectrum is characterized by an intense reflectance peak spanning from about 500 to 620 nm, centered at 550 nm and assigned to the stop-band. On the red-edge side of the stop-band, at 570 nm it is evident a relative minimum, assigned to a cavity mode induced by the presence of the

defect layer. The reflectance measured after 60 days (black dashed line) demonstrates the stability of the structure quality, maintaining permanently high reflectivity. The emission of the dye is strongly modified by the dielectric environment (Figure S12 c). The pristine F8BT film shows indeed a broad photoluminescence signal ranging from 470 nm to 700 nm and peaked at 540 nm. Once inserted in the cavity, the emission is redistributed - suppressed at wavelengths corresponding to the stop-band and enhanced 4-fold at the cavity mode. The emission spectrum from the microcavity has the highest intensity at 574 nm and a shoulder at 586 nm. The finesse of the structure is quantified by the quality factor, that is the ratio between cavity mode wavelength and its full width half maximum, as Q=60, a remarkable value for a hybrid structure.[4] Moreover, by collecting the emission intensity at different angles it is possible to appreciate the angular dispersion of the cavity mode (Figure S13 d, emission intensity in color code). The cavity mode continuously shifts from 574 nm at normal collection and reaches 530 nm at about 45 degrees. At higher angles, the emission is not affected anymore by the environment, in agreement with theory.[4]

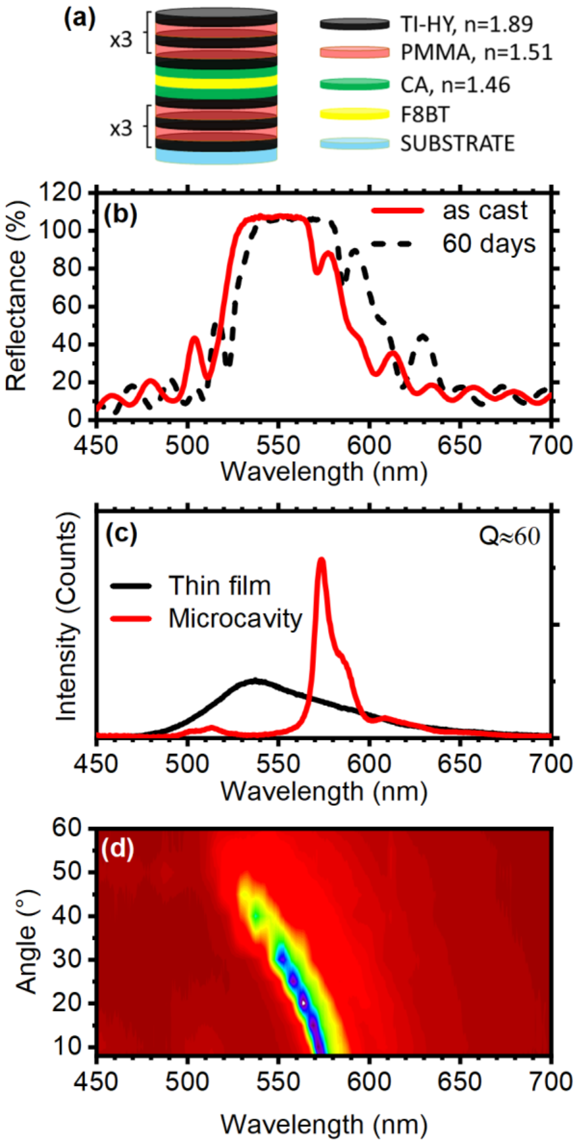

Figure S14. (a) Structure of a Ti-Hy:PMMA microcavity made of two DBRs of 14.5 periods sandwiching a layer of F8BT (b) Reflectance spectra of the structure as cast(red line) and after 60 days (black dashed line). (c) Photoluminescence spectrum of a pristine F8BT film and of an identical one placed in a microcavity. (d) Dispersion of the emission intensity form the microcavity with collection angle.

Figure S15 shows the absorbance spectra of the multilayered structures in a solution of methylene blue with an initial concentration of 6 ppm (black lines) and after irradiation with a 360 nm light emitting diode for set time intervals. All the spectra reveal the presence of the dye and a decrease in intensity with the irradiation time.

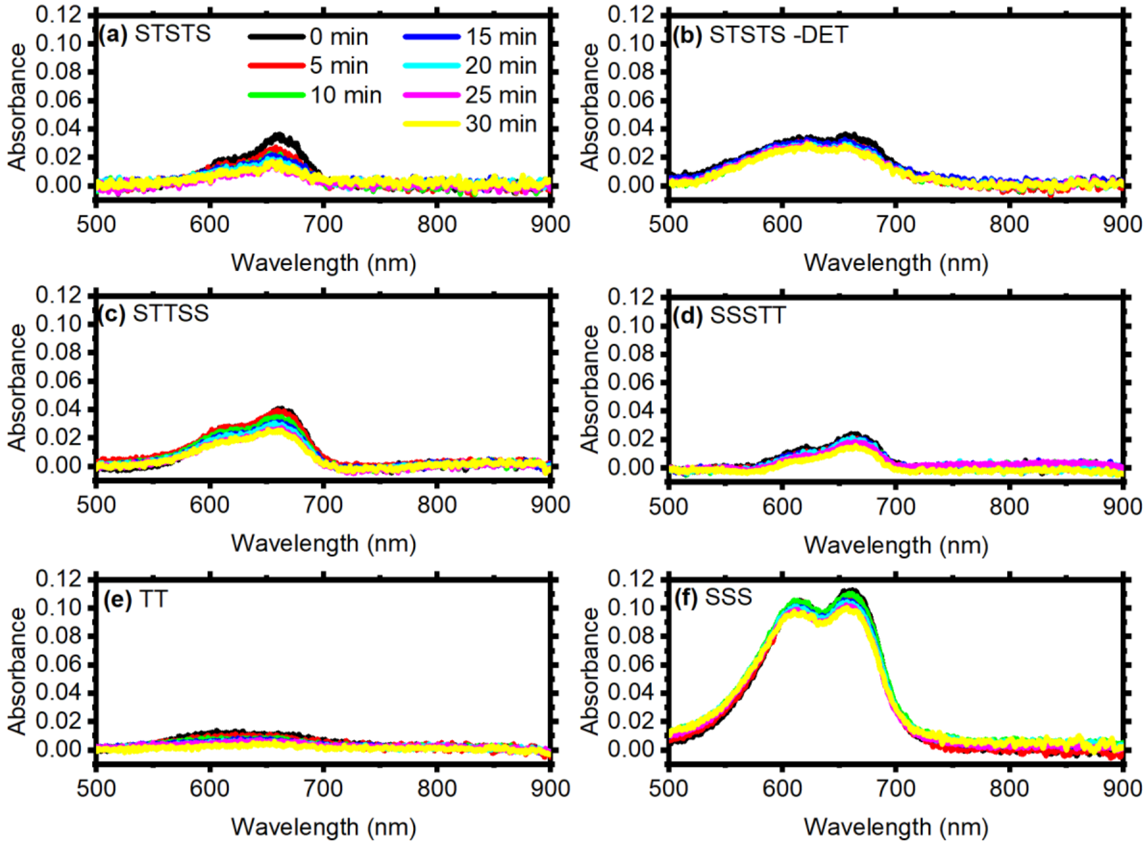

Figure S15. Methylene blue absorbance in the multilayered structures: (a) STSTS; (b) STSTS-DET, (c) STTSS, (d) SSSTT, (e) TT; (f) SSS.

Effect of refractive index variation in the DBR porosity

The spectral position of the stop-band center (λ_{PBG}) in a DBR can be described in a simple way by the Bragg-Snell law [4]

$$\lambda_{PBG} = 2D\sqrt{n_{eff}^2 - sin^2(\theta)} \quad \text{(S1)}$$

where n_{eff} is the effective refractive index of the structure, D is the lattice pitch, and θ the incidence angle of light. n_{eff} is the average refractive index of composing materials weighted over their volume fraction, that for a monodimensional structure is expressed by the thickness of the layers. Being $D = d_{Ti-Hy} + d_{Si-Hy}$, it is easy to calculate n_{eff} within the Maxwell-Garnett approximation as:[4, 7]

$$n_{eff} = \sqrt{n_{Ti-Hy}^2 \frac{d_{Ti-Hy}}{D} + n_{Si-Hy}^2 \frac{d_{Si-Hy}}{D}} \quad \text{(S2)}$$

To describe the effect on the solvent intercalating into the lattice porosity, a further effective medium for the Ti-Hy and the Si-Hy is necessary. Indeed, each hybrid layer is constituted by the inorganic part, a negligible amount of PAA (see Table 1) and voids. We can then apply a simplified effective medium model applying the Maxwell-Garnett approximation to the single layers,[7] considering the analyte (a) intercalating into the void. The effective refractive index of the DBR becomes:

$$n_{eff} = \sqrt{\left[n_{TiO_2}^2\, f_{TiO_2} + \left(1 - f_{TiO_2}\right) n_A^2\right] \frac{d_{Ti-Hy}}{D} + \left[n_{SiO_2}^2\, f_{SiO_2} + \left(1 - f_{SiO_2}\right) n_A^2\right] \frac{d_{Si-Hy}}{D}} =$$

$$\sqrt{n_{TiO_2}^2 f_{TiO_2} \frac{d_{Ti-Hy}}{D} + n_{SiO_2}^2 f_{SiO_2} \frac{d_{Si-Hy}}{D} + \frac{n_A^2}{D}\left[\left(1 - f_{TiO_2}\right) d_{Hy-Ti} + \left(1 - f_{SiO_2}\right) d_{Si-Hy}\right]} = \quad \text{(S3)}$$

$$\sqrt{\frac{A^2}{D} + \frac{n_A^2}{D}\left[\left(1 - f_{TiO_2}\right) d_{Ti-Hy} + \left(1 - f_{SiO_2}\right) d_{Si-Hy}\right]} = \frac{A}{\sqrt{D}}\sqrt{1 + \frac{n_A^2 B^2}{A^2}}$$

where

$$A^2 = n_{TiO_2}^2 f_{TiO_2} d_{Ti-Hy} + n_{SiO_2}^2 f_{SiO_2} d_{Si-Hy} \tag{S5}$$

and

$$B^2 = \left(1 - f_{TiO_2}\right) d_{Ti-Hy} + \left(1 - f_{SiO_2}\right) d_{Si-Hy} \tag{S6}$$

References

(1) Hsu, L. S.; Rujkorakarn, R.; Sites, J. R.; She, C. Y., Thermally Induced Crystallization of Amorphous‐Titania Films, *J. Appl. Phys.*, **1986**, *59*, 3475-3480.

(2) Sarkar, S.; Gupta, V.; Kumar, M.; Schubert, J.; Probst, P. T.; Joseph, J.; König, T. A. F., Hybridized Guided-Mode Resonances via Colloidal Plasmonic Self-Assembled Grating, *ACS Appl. Mater. Intefaces*, **2019**, *11*, 13752-13760.

(3) Rodríguez-de Marcos, L. V.; Larruquert, J. I.; Méndez, J. A.; Aznárez, J. A., Self-Consistent Optical Constants of SiO_2 and Ta_2O_5 films, *Opt. Mater. Express*, **2016**, *6*, 3622-3637.

(4) Lova, P.; Manfredi, G.; Comoretto, D., Advances in Functional Solution Processed Planar One-Dimensional Photonic Crystals, *Adv. Opt. Mater.*, **2018**, *6*, 1800730-26.

(5) Sultanova, N.; Kasarova, S.; Nikolov, I., Dispersion Properties of Optical Polymers, *Acta Phys. Pol.*, **2009**, *116*.

(6) Lova, P.; Bastianini, C.; Giusto, P.; Patrini, M.; Rizzo, P.; Guerra, G.; Iodice, M.; Soci, C.; Comoretto, D., Label-Free Vapor Selectivity in Poly(p-Phenylene Oxide) Photonic Crystal Sensors, *ACS Applied Materials & Interfaces*, **2016**, *8*, 31941-31950.

(7) Gehr, R. J.; Boyd, R. W., Optical Properties of Nanostructured Optical Materials, *Chem. Mater.*, **1996**, *8*, 1807.
